# Air-stable aryl derivatives of pentafluoroorthotellurate†

**DOI:** 10.1039/d2cc03936b

**Published:** 2022-08-08

**Authors:** Daniel Wegener, Kurt F. Hoffmann, Alberto Pérez-Bitrián, Ilayda Bayindir, Amiera N. Hadi, Anja Wiesner, Sebastian Riedel

**Affiliations:** Fachbereich Biologie, Chemie, Pharmazie, Institut für Chemie und Biochemie – Anorganische Chemie, Freie Universität Berlin Fabeckstraße 34/36 14195 Berlin Germany s.riedel@fu-berlin.de

## Abstract

We report on two different sets of air-stable derivatives of pentafluoroorthotellurate containing fluorinated and non-fluorinated aryl groups. The acid *cis*-PhTeF_4_OH was obtained in gram scale and further transformed to Ag[*cis*-PhTeF_4_O], which was used as a *cis*-PhTeF_4_O transfer reagent to obtain [PPh_4_][*cis*-PhTeF_4_O]. Furthermore, the synthesis of *trans*-(C_6_F_5_)_2_TeF_3_OH was achieved by a selective hydrolysis of *trans*-(C_6_F_5_)_2_TeF_4_ in the presence of KF and subsequent protonation by *a*HF. Quantum-chemical calculations show a higher acidity and robustness against fluoride abstraction for *trans*-(C_6_F_5_)_2_TeF_3_OH compared to *cis*-PhTeF_4_OH.

## Abstract 2

The synthesis of air-stable aryl derivatives of OTeF__5__ has been achieved through the use of convenient procedures. Pentafluorophenyl derivatives are stronger acids and more robust against electrophiles in comparison to phenyl analogues.

## Graphical abstract

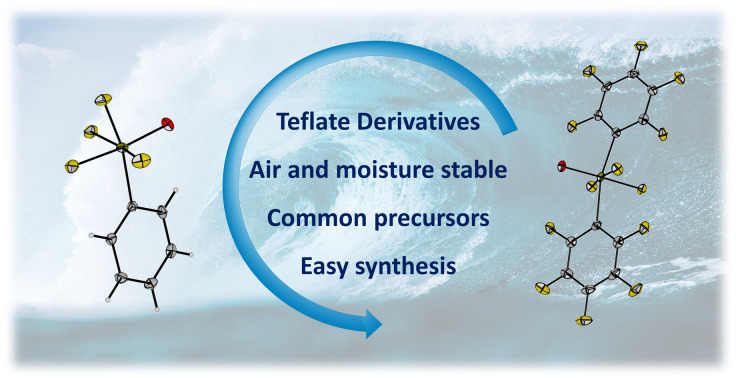

The pentafluoroorthotellurate group (teflate, OTeF_5_) is known for its robustness against oxidizers and electrophiles, the high delocalization of the negative charge, and its strong electron withdrawing properties, which are comparable to fluorine.^1,2^ As a consequence, the teflate group provides access to a variety of weakly coordinating anions (WCAs),^3–5^ strong Lewis acids^5^ and highly reactive species.^1–3,6^ Nevertheless, compounds containing teflate groups also present some drawbacks, which include the sensitivity to hydrolysis leading to the formation of degradation products (*e.g.* HF).^1^ Additionally, the synthesis of HOTeF_5_, which is the most commonly used teflate source, requires special starting materials (Te(OH)_6_ and HSO_3_F).^7,8^ These properties severely limit the potential field of application of the OTeF_5_ group and only allow the work under strictly inert conditions.

During the last years (perfluoro)organotellurium chemistry has expanded significantly.^9,10^ The substitution of some of the fluorine atoms in the OTeF_5_ group by (perfluoro)aryl groups might help overcome its instability towards water.^11^ The access to organotellurium(vi) fluorides has been historically achieved through the oxidative fluorination of organotellurium compounds in lower oxidation states. However, this required the use of harsh conditions and strong oxidizers, leading in most cases to low yields or impure reaction mixtures, which could not be always isolated.^12–14^ For example, the oxidation of Te(C_6_F_5_)_2_ with elemental fluorine led to (C_6_F_5_)_2_TeF_2_ and *cis*-(C_6_F_5_)_2_TeF_4_ stepwise, but faced the problem of further fluorination of the aryl rings.^12^ On the other hand, PhTeF_5_ was prepared *via* oxidation of Ph_2_Te_2_ with XeF_2_ and, although it was of great interest for reactivity studies,^15–17^ it was never isolated prior to use.^13–17^

Recently, a new system for the oxidative fluorination of diarylditellurides consisting of trichloroisocyanuric acid (TCICA), potassium fluoride and catalytic amounts of trifluoroacetic acid has been reported.^18^ This system has been successfully applied to the synthesis of a broad scope of TeF_5_-substituted arenes in good yields, which include the PhTeF_5_ derivative. The easy access to these compounds has therefore allowed the study of the properties and reactivity of the TeF_5_ moiety.^11,18,19^ Compound PhTeF_5_ was found to be an air-stable molecule, in contrast to previous descriptions, yet could be hydrolysed quantitatively to *cis*-PhTeF_4_OH in a mixture of acetonitrile and water.^18^

Herein we report on the synthesis and characterization of two different aryl-substituted derivatives of the pentafluoroorthotellurate, namely [*cis*-PhTeF_4_O]^−^ and [*trans*-(C_6_F_5_)_2_TeF_3_O]^−^,^20^ and their corresponding Brønsted acids.

Compound *cis*-PhTeF_4_OH (1) was prepared through a modified procedure (see ESI† for details).^18^ It was obtained as a colourless oil in gram scale and in excellent yield (Scheme 1). The *cis* arrangement of the phenyl and hydroxy substituents is observed both in solution and in the solid state, as confirmed by ^19^F NMR spectroscopy and single-crystal X-ray diffraction, respectively. Compound 1 represents the first crystallized species containing the TeF_4_OH moiety connected to an aryl and can be considered as a derivative of HOTeF_5_. The Brønsted acid *cis*-PhTeF_4_OH crystallizes in the orthorhombic space group *Pbca* and shows a distorted octahedral arrangement at the Te centre (Fig. 1), with C–Te–O angles of 93.9(1)° and 98.6(1)° and Te–F bonds between 184.8(2) and 187.4(2) pm. Two independent molecules are found in the asymmetric unit, which are connected through a hydrogen bond. The O⋯O distance is 270.7 pm, therefore being in the expected range for such non-covalent interaction.^21^

**Scheme 1:**
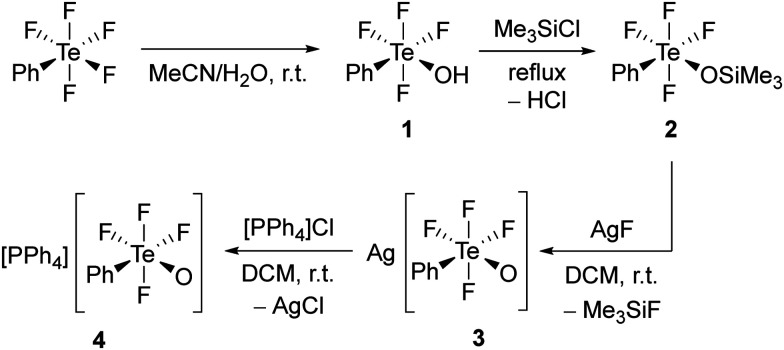
Synthesis of compounds containing the *cis*-PhTeF_4_O moiety.

**Fig. 1:**
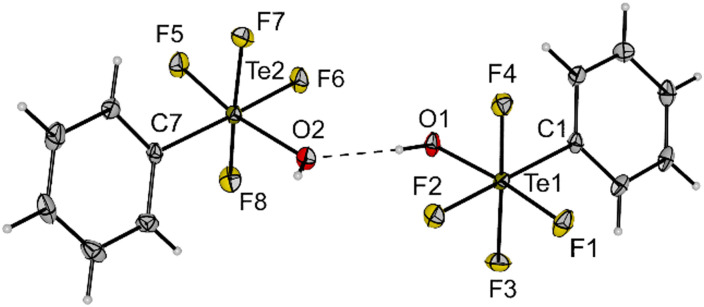
Molecular structure of *cis*-PhTeF_4_OH (1) in the solid state. Displacement ellipsoids set at 50% probability. Selected bond lengths [pm] and angles [°]: Te1–F1 187.3(2), Te1–F2 185.5(2), Te1–F3 186.6(2), Te1–F4 187.0(2), Te1–O1 189.7(2), Te1–C1 207.2(3), O1–Te1–F1 172.0(1), C1–Te1–F2 178.2(1), F3–Te1–F4 170.5(1), C1–Te1–O1 93.9(1), Te2–F5 187.4(2), Te2–F6 184.8(2), Te2–F7 186.9(2), Te2–F8 186.4(2), Te2–O2 189.7(2), Te2–C7 207.8(3), O1···O2 270.7, O2–Te2–F5 167.8(1), C7–Te2–F6 178.1(1), F7–Te2–F8 170.4(1), C7–Te2–O2 98.6(1). For crystallographic details see ESI.†

The ^19^F NMR spectrum of 1 shows the characteristic ABC_2_X spin system with signals at −25.8, −47.0, and −50.5 ppm and corresponding ^2^*J*(^19^F,^19^F) coupling constants of 148, 134, and 109 Hz. The NMR spectroscopic data are in agreement with those previously reported by Togni *et al.*,^18^ and are also comparable with the signals reported for *cis*-PhTeF_4_Cl.^22^

Contrary to the synthesis of [N(*n*-Bu)_4_][OTeF_5_], which is readily prepared from HOTeF_5_ and [N(*n*-Bu)_4_]Cl,^8^ compound 1 does not react with a comparable chloride salt such as [PPh_4_]Cl. In order to increase the reactivity and achieve the transfer of a *cis*-PhTeF_4_O group, we aimed at preparing the silver salt of the [*cis*-PhTeF_4_O]^−^ anion and using the formation of poorly soluble silver halides as the driving force of the corresponding reaction. With this objective, *cis*-PhTeF_4_OH (1) was reacted with Me_3_SiCl to form *cis*-PhTeF_4_OSiMe_3_ (2), which could be subsequently transformed into Ag[*cis*-PhTeF_4_O] (3) selectively (see Scheme 1). The preparation of *cis*-PhTeF_4_OSiMe_3_ (2) from *cis*-PhTeF_4_OH (1) was carried out according to a literature procedure reported for related compounds.^23^

The transformation of *cis*-PhTeF_4_OSiMe_3_ (2) into Ag[*cis*-PhTeF_4_O] (3) was achieved by reaction with AgF in dichloromethane, whereby the stable and volatile Me_3_SiF was formed. The reaction reaches full conversion after 16 h at room temperature. The ^19^F NMR spectra of 2 and 3 show similar patterns in agreement with the ABC_2_X spin system. The salt Ag[*cis*-PhTeF_4_O] (3) is poorly soluble in many organic solvents, but moderately in acetonitrile, although the solubility increases significantly by the addition of pyridine.

Compound 3 is a suitable transfer reagent of the air-stable *cis*-PhTeF_4_O group, as it was shown by its reaction with [PPh_4_]Cl in dichloromethane. The resulting salt [PPh_4_][*cis*-PhTeF_4_O] (4) was isolated as a colourless solid in 92% yield after filtration to separate the formed AgCl. Single crystals of [PPh_4_][*cis*-PhTeF_4_O] grew in the monoclinic space group *P*2_1_/*c*. The molecular structure in the solid state represents the first example of a structurally characterized [RTeF_4_O]^−^ anion (R = aryl). The [*cis*-PhTeF_4_O]^−^ anion possesses a distorted octahedral arrangement at the Te centre (Fig. 2) with marginally longer Te–F (189.3(1)–190.7(1) pm) and Te–O (179.8(2) pm) bonds than in the teflate anion (Te–F: 184.6–186.2 pm; Te–O: 178.9 pm).^24^ The ^19^F NMR spectrum shows the previously mentioned typical pattern for such derivatives.

**Fig. 2:**
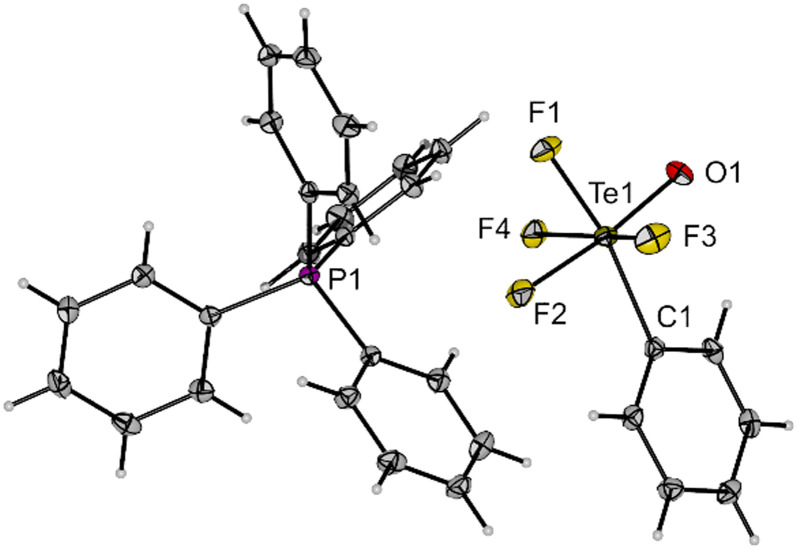
Molecular structure of [PPh_4_][*cis*-PhTeF_4_O] (4) in the solid state. Displacement ellipsoids set at 50% probability. Selected bond lengths [pm] and angles [°]: Te1–F1 189.3(1), Te1–F2 190.5(1), Te1–F3 190.1(1), Te1–F4 190.7(1), Te1–O1 179.8(2), Te1–C1 210.5(2), O1–Te1–F2 173.7(1), C1–Te1–F1 167.4(1), F3–Te1–F4 165.4(1), C1–Te1–O1 99.1(1). For crystallographic details see ESI.†

To determine the robustness against fluoride abstraction of the [*cis*-PhTeF_4_O]^−^ anion in the context of different teflate derivatives, the fluoride ion affinities (FIA) of the corresponding neutral oxo compounds have been calculated (see Table 1). As expected, fluorination of the aryl group increases the fluoride ion affinity (FIA), therefore meaning that the [*cis*-(C_6_F_5_)TeF_4_O]^−^ and [*trans*-(C_6_F_5_)_2_TeF_3_O]^−^ anions possess Te–F bonds that are less prone to fluoride abstraction. Additionally, the proton affinities of the corresponding bases also increase upon fluorination of the aryl groups (see Table 1), which shows that the corresponding Brønsted acid is more suitable for protonation reactions aiming at the transfer of the teflate derivative. Both trends prompted us to develop a synthetic route to access a derivative containing C_6_F_5_ groups.

**Table 1:** Calculated fluoride ion affinities (FIA) and proton affinities (PA) of teflate and its derivatives^a^

System	FIA^b^/kJ mol^−1^	PA^c^/kJ mol^−1^
OTeF_4_/[OTeF_5_]^−^	454	1281
PhTeF_3_O/[*cis*-PhTeF_4_O]^−^	349	1352
Ph_2_TeF_2_O/[*trans*-Ph_2_TeF_3_O]^−^	294	1388
(C_6_F_5_)TeF_3_O/[*cis*-(C_6_F_5_)TeF_4_O]^−^	407	1302
(C_6_F_5_)_2_TeF_2_O/[*trans*-(C_6_F_5_)_2_TeF_3_O]^−^	368	1308

a *Trans* refers to the relative arrangement of the aryl rings (*cf.*5).

b DFT calculations performed on BP86/def-SV(P) level of theory. Isodesmic reactions with Me_3_SiF/Me_3_Si^+^ as anchor were used.^25^

c DFT calculations performed on B3LYP/def2-TZVPP level of theory.

The aforementioned TCICA/KF oxidation system was applied to the fluorination of Te(C_6_F_5_)_2_, leading to the isolation of *trans*-(C_6_F_5_)_2_TeF_4_ (5) as a white solid in 85% yield (Scheme 2). In contrast, fluorination with elemental fluorine at low temperature leads to *cis*-(C_6_F_5_)_2_TeF_4_ quantitatively, as described by Naumann *et al.* in 1985.^12^ Our reaction represents the first application of the TCICA/KF oxidation system to a telluride with two perfluorinated substituents, although the use of this protocol with diorganyl monotellurides has been preliminary demonstrated.^18,26^ More interestingly, it shows the tolerance of the system to compounds with *ortho*-substituted aromatics, in contrast to previous assumptions.^18^

**Scheme 2:**
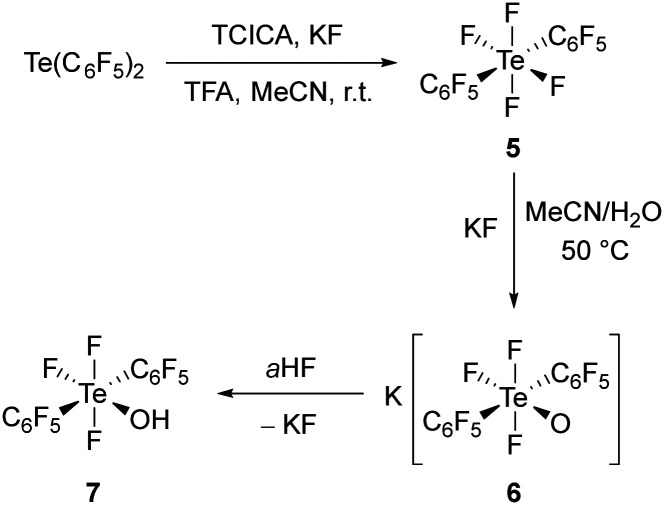
Synthesis of compounds containing the *trans*-(C_6_F_5_)_2_TeF_3_O moiety.

The *trans* arrangement of the two C_6_F_5_ rings at the Te centre is easily assigned from the ^19^F NMR spectrum, as only one resonance for the equatorial fluorine atoms is observed. It appears as a quintet at a chemical shift of −21.4 ppm, with coupling constants of ^4^*J*(^19^F,^19^F_*o*_) = 19 Hz and ^1^*J*(^19^F,^125^Te) = 3104 Hz. The ^125^Te NMR spectrum shows a quintet of multiplets at 770 ppm due to coupling of the tellurium to the four directly bound equatorial F, as well as to the fluorine atoms of the aromatic rings.

Single crystals of 5 suitable for X-ray diffraction were grown from a *n*-hexane solution of the compound at −40 °C. The compound crystallizes in the orthorhombic space group *Pbca* and the molecular structure in the solid state shows the same *trans* arrangement of the C_6_F_5_ groups as in solution (Fig. 3). The molecule contains an octahedrally coordinated Te centre with Te–F bond lengths (186.4(1) and 187.2(1) pm) similar to 1 (*cf.*Fig. 1). Compound 5 is a moisture- and air-stable compound, rather unreactive and soluble in different organic solvents (*e.g. n*-hexane, acetonitrile, DCM, THF, *o*-DFB), which makes it an excellent precursor for the synthesis of a teflate derivative with improved properties.

**Fig. 3:**
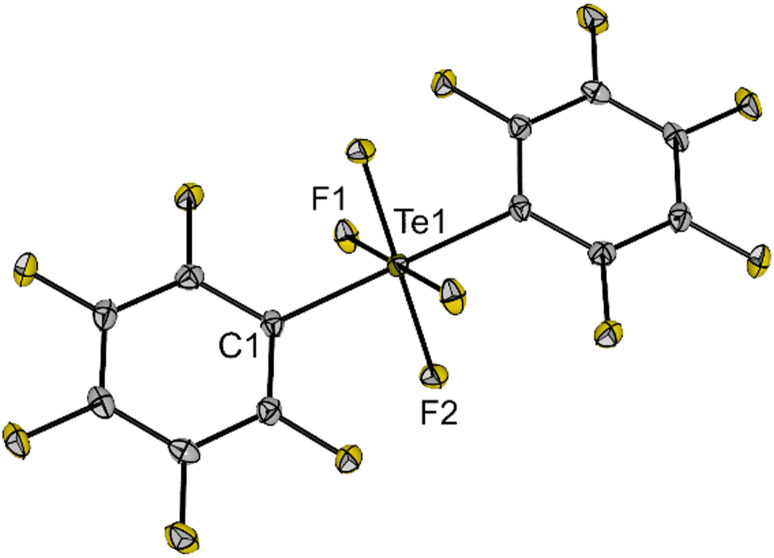
Molecular structure of *trans*-(C_6_F_5_)_2_TeF_4_ (5) in the solid state. Displacement ellipsoids set at 50% probability. Selected bond lengths [pm] and angles [°]: Te1–F1 187.2(1), Te1–F2 186.4(1), Te1–C1 208.7(2), C1–Te1–F1 89.7(1), C1–Te1–F2 90.3(1), F1–Te1–F2 89.4(1). For crystallographic details see ESI.†

Due to the equivalent nature of the four Te–F bonds in *trans*-(C_6_F_5_)_2_TeF_4_ (5), the selective hydrolysis of only one of them might be challenging. In fact, by using the same hydrolysis protocol as for PhTeF_5_, no reaction was observed and, unfortunately, heating the reaction mixture to 50 °C led to mixtures containing the doubly hydrolysed species (see ESI†). The functionalization of one Te–F bond of 5 by using Me_3_SiOMe or Me_3_SiNMe_2_, similarly to Janzen *et al.*,^17^ was also tested, yet no reaction was observed either. Gratifyingly, we found that the hydrolysis of the second Te–F bond can be prevented if KF is added to the water/acetonitrile mixture. Following this protocol, K[*trans*-(C_6_F_5_)_2_TeF_3_O] (6) was obtained in excellent yield (see Scheme 2). The hydrolysis of 5 proceeds with retention of the *trans* arrangement of the C_6_F_5_ substituents. This is observed in the ^19^F NMR spectrum of 6, which shows a triplet of quintets (*δ* = 32.3 ppm) and a doublet of quintets (*δ* = −18.8 ppm), with an integral ratio of 1 : 2, accounting for the two chemically inequivalent ^19^F nuclei (^2^*J*(^19^F,^19^F) = 104 Hz). Additional splitting is present due to the coupling to the F_o_ of the C_6_F_5_ rings (^4^*J*(^19^F,^19^F_*o*_) ≈ 20 Hz).

Single crystals of K[*trans*-(C_6_F_5_)_2_TeF_3_O]·MeCN were obtained by slow diffusion of diethyl ether into an acetonitrile solution of 6 and were examined by X-ray diffraction. The salt crystallizes in the monoclinic space group *P*2_1_/*c* and the Te centre in the [*trans*-(C_6_F_5_)_2_TeF_3_O]^−^ anion adopts a distorted octahedral geometry with the expected *trans* arrangement of the C_6_F_5_ units (Fig. 4). The C1–Te1–C7 angle is 166.3(1)° and thus, deviates significantly from the ideal angle of 180°. The Te–F (189.8(1)–191.2(1) pm) and Te–O bonds (178.0(1) pm) have approximately the same length as in the [*cis*-PhTeF_4_O]^−^ anion (*cf.*Fig. 2). The molecular structure in the solid state shows a K⋯O interaction with a distance of 258.8(1) pm. Furthermore, the potassium cation is coordinated by one acetonitrile molecule. The salt K[*trans*-(C_6_F_5_)_2_TeF_3_O] (6) represents a moisture- and air-stable compound, which shows high solubility in different organic solvents (*e.g.* toluene, acetonitrile, THF, *o*-DFB).

**Fig. 4:**
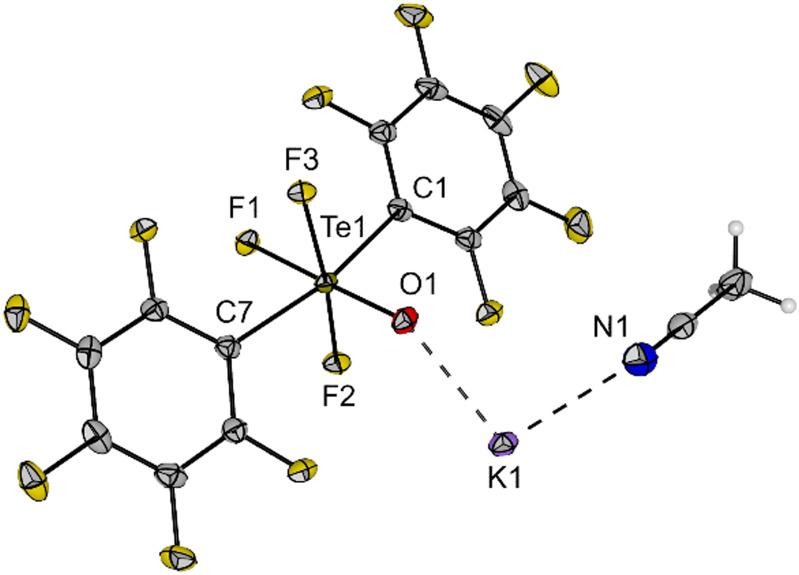
Molecular structure of K[*trans*-(C_6_F_5_)_2_TeF_3_O] (6) in the solid state. Displacement ellipsoids set at 50% probability. Selected bond lengths [pm] and angles [°]: Te1–F1 191.1(1), Te1–F2 191.2(1), Te1–F3 189.8(1), Te1–O1 178.0(1), Te1–C1 213.5(2), Te1–C7 212.4(2), K1–O1 258.8(1), O1–Te1–F1 178.9(1), F2–Te1–F3 170.7(1), C1–Te1–C7 166.3(1). For crystallographic details see ESI.†

The need for a fluoride source during the hydrolysis of 5 led to the formation of the anionic [*trans*-(C_6_F_5_)_2_TeF_3_O]^−^, instead of the corresponding acid, in contrast to the synthesis of 1. However, when 6 is treated with *a*HF, quantitative formation of *trans*-(C_6_F_5_)_2_TeF_3_OH (7) takes place. The acid 7 was isolated after extraction in dichloromethane in 77% yield as a hygroscopic solid. In the ^19^F NMR spectrum, the triplet of quintets is high-field shifted by approx. 30 ppm, compared to the corresponding signal in the ^19^F NMR spectrum of K[*trans*-(C_6_F_5_)_2_TeF_3_O] (6). Moreover, the coupling constant between the fluorine atoms directly bound to the tellurium centre decreases to ^2^*J*(^19^F,^19^F) = 54 Hz. The protonation becomes also clear from the ^1^H NMR spectrum, which shows the resonance of the proton as a broad singlet at 5.76 ppm. The IR spectrum of the compound also shows a broad band at 3493 cm^−1^, which is due to the O–H stretching vibration. Furthermore, in the ESI mass spectrum the signal of the dimer [((C_6_F_5_)_2_TeF_3_O)_2_H]^−^ can be observed at *m*/*z* = 1070.7.

In conclusion, we developed the synthesis of two different sets of derivatives of the pentafluoroorthotellurate containing aryl substituents. The Brønsted acid *cis*-PhTeF_4_OH (1) could be obtained in gram scale and was transformed into the corresponding silver salt Ag[*cis*-PhTeF_4_O] (3), which is a suitable transfer reagent of the *cis*-PhTeF_4_O group. Its reactivity was demonstrated by the synthesis of [PPh_4_][*cis*-PhTeF_4_O] (4) starting from [PPh_4_]Cl. Furthermore, we showed the facile synthesis of *trans*-(C_6_F_5_)_2_TeF_4_ (5) and its hydrolysis in the presence of KF to form K[*trans*-(C_6_F_5_)_2_TeF_3_O] (6), which could be subsequently protonated with *a*HF to yield the Brønsted acid *trans*-(C_6_F_5_)_2_TeF_3_OH (7). Additionally, calculations showed that fluorination of the phenyl rings leads not only to a higher robustness towards fluoride abstraction, but also increases the strength of the corresponding Brønsted acids. Therefore, these compounds are moisture- and air-stable analogues of the teflate, which can be easily obtained with cheap starting materials and through simple procedures. Due to these outstanding properties, both *cis*-PhTeF_4_O and *trans*-(C_6_F_5_)_2_TeF_3_O arise as promising groups for the synthesis of a new family of WCAs and Lewis acids.

Funded by the Deutsche Forschungsgemeinschaft (DFG, German Research Foundation)—Project-ID 387284271—SFB 1349 and the ERC Project HighPotOx. Computing time was made available by the High-Performance Computing Center at the ZEDAT, Freie Universität Berlin. We gratefully acknowledge the assistance of the Core Facility BioSupraMol supported by the DFG. A. P.-B. thanks the Alexander von Humboldt Foundation for a postdoctoral research fellowship.

## Conflicts of interest

There are no conflicts to declare.

## Supplementary Material

CC-058-D2CC03936B-s001

CC-058-D2CC03936B-s002
