# Supplementary material for: Air-stable aryl derivatives of pentafluoroorthotellurate
Source: Chem Commun (Camb). 2022 Aug 8;58(69):9694–7. doi: 10.1039/d2cc03936b (PMC9404409; doi:10.1039/d2cc03936b)
Supplement: CC-058-D2CC03936B-s001 [file CC-058-D2CC03936B-s001.pdf]

Electronic Supplementary Material (ESI) for ChemComm.
This journal is © The Royal Society of Chemistry 2022

Supporting Information

for

Air-stable Aryl Derivatives of the Pentafluoroorthotellurate

Daniel Wegener, Kurt F. Hoffmann, Alberto Pérez-Bitrián, Ilayda Bayindir, Amiera N. Hadi, Anja Wiesner and Sebastian Riedel

Table of contents

1. Experimental section S2
2. NMR spectra S8
3. IR spectra S15
4. Crystal data S17
5. Attempted hydrolysis of *trans*-$(C_6F_5)_2TeF_4$ (**5**) S22
6. Quantum-chemical calculations S23
7. References S31

1 Experimental section

General procedures and materials

Unless otherwise mentioned, all experiments were performed under exclusion of moisture and oxygen using standard Schlenk techniques. Solids were handled in a MBRAUN UNIlab plus glovebox under an argon atmosphere (O_2 < 0.5 ppm, H_2O < 0.5 ppm). All experiments involving anhydrous HF (*a*HF) were performed in self-built PFA (perfluoroalkoxy alkanes) tubes connected to stainless steel metal valves and with a stainless steel vacuum line. Solvents were dried using a MBraun SPS-800 solvent system (CH_2Cl_2, MeCN, *n*-pentane), or with CaH_2 (*o*-DFB, Et_2O, CD_3CN, CD_2Cl_2) before use and stored over 3 or 4 Å molecular sieves. $PhTeF_5$ and $Te(C_6F_5)_2$ were prepared according to literature procedures.[1,2] All other reagents were purchased from standard commercial suppliers and used as received. NMR spectra were recorded on a JEOL 400 MHz ECS or JEOL 400 MHz ECZ spectrometer. All reported chemical shifts (*δ* in ppm) are referenced to the Ξ values given in IUPAC recommendations of 2008 using the ^2H signal of the deuterated solvent as internal reference.[3] Multiplicity is indicated as follows: s = singlet, t = triplet, quint = quintet, dd = doublet of doublets, dt = doublet of triplets, dquint = doublet of quintets, tquint = triplet of quintets, m = multiplet. IR spectra were measured at room temperature on a Bruker ALPHA FTIR spectrometer with a diamond ATR inside a glovebox under an argon atmosphere, or on a Nicoleti S50 Advance FTIR by Thermo Fisher Scientific equipped with an ATR unit, with a Ge on KBr beam splitter and a DLaTGS-KBr detector for MIR and a solid-substrate beam splitter with a DLaTGS-PE detector for FIR. The ESI-TOF-Mass spectrometry measurements were performed on an Agilent 6210 ESI-TOF, Agilent Technologies, Santa Clara, CA, USA. Solvent flow rate was adjusted to 4 µL/min, spray voltage set to 4 kV. Drying gas flow rate was set to 15 psi (1 bar). Elemental analyses (CHNS) were carried out using a VARIO EL elemental analyzer. Crystal data were collected with MoKα radiation on a Bruker D8 Venture diffractometer with a CMOS area detector. Single crystals were picked −40 °C under nitrogen atmosphere and mounted on a 0.15 mm Mitegen micromount using perfluoroether oil. The structures were solved with the ShelXT[4] structure solution program using intrinsic phasing and refined with the ShelXL[5] refinement package using least squares minimizations by using OLEX2.[6] For visualization the program Diamond V4.6.4 was used.[7] CCDC 2184677, 2184678, 2184711, 2184734 and 2184735 contain the supplementary crystallographic data for this paper. These data are provided free of

charge by The Cambridge Crystallographic Data Centre. Crystal data and other details of the structure analyses are summarized in Tables S1–S5. Suitable crystals for X-ray diffraction studies were obtained as indicated in the corresponding experimental entry (*vide infra*).

Synthesis of *cis*-$PhTeF_4OH$ (**1**)

$PhTeF_5$ (3.70 g, 12.3 mmol) was dissolved in a $MeCN/H_2O$ mixture (9:1 V/V, 150 mL) and stirred at room temperature for 20 min. CH_2Cl_2 (5 mL) and H_2O (10 mL) were added to the obtained solution and the resulting phases were separated. The aqueous phase was extracted with CH_2Cl_2 (3×10 mL). The combined organic phases were dried with $MgSO_4$, filtered, and the solvent was removed under reduced pressure. A yellow oil was obtained and characterized as compound **1** (3.65 g, 12.3 mmol, 99% yield). Single crystals of **1** suitable for X-ray diffraction were obtained by cooling a saturated solution of **1** in *n*-pentane to –40 °C.

^{1}H NMR (400 MHz, CD_2Cl_2, 23 °C): δ = 7.93–7.90 (m, 2H, $^3J(^1H,^1H)$ = 7.7 Hz, *o*-H), 7.76–7.71 (m, 1H, $^3J(^1H,^1H)$ = 7.6 Hz, *p*-H), 7.71–7.66 (m, 2H, $^3J(^1H,^1H)$ = 7.6 Hz, *m*-H), 5.56 (br s, OH) ppm.

^{19}F NMR (377 MHz, CD_2Cl_2, 23 °C): δ = –25.8 (dt, 1F, $^2J(^{19}F,^{19}F)$ = 148; 134 Hz, $^1J(^{125}Te,^{19}F)$ = 2860 Hz), –47.0 (m, 1F, $^2J(^{19}F,^{19}F)$ = 134; 109 Hz, $^1J(^{125}Te,^{19}F)$ = 3374 Hz), –50.5 (m, 2F, $^2J(^{19}F,^{19}F)$ = 134; 109 Hz, $^1J(^{125}Te,^{19}F)$ = 3353 Hz) ppm.

IR (ATR, 25 °C, Figure S14): $\tilde{\nu}$ = 3502 (m, O−H), 3067 (w, C−H), 1480 (m, Ph-ring), 1448 (m, Ph-ring), 997 (m), 928 (m), 734 (m), 673 (s, O−Te−F), 630 (s, Te−F), 454 (s) cm^{-1}.

Synthesis of *cis*-$PhTeF_4OSiMe_3$ (**2**)

cis-$PhTeF_4OH$ (1.40 g, 4.70 mmol) was placed in a Schlenk flask and cooled to –196°C. Me_3SiCl (1.02 g, 9.40 mmol) was condensed onto it and the reaction mixture was heated at 60 °C for 5 h. After removal of the volatiles under reduced pressure, a yellow oil was obtained and characterized as **2** (1.59 g, 4.30 mmol, 91% yield).

^{1}H NMR (400 MHz, CD_2Cl_2, 23 °C): δ = 7.91-7.87 (m, 2H, $^{3}J(^{1}H,^{1}H)$ = 7.7 Hz, *o*-H), 7.75-7.70 (m, 1H, $^{3}J(^{1}H,^{1}H)$ = 7.3 Hz, *p*-H), 7.69-7.62 (m, 2H, $^{3}J(^{1}H,^{1}H)$ = 7.9 Hz, *m*-H), 0.3 (s, 9H, CH_3) ppm.

^{19}F NMR (377 MHz, CD_2Cl_2, 23 °C): δ = –23.2 (dt, 1F, $^{2}J(^{19}F,^{19}F)$ = 154; 134 Hz, $^{1}J(^{125}Te,^{19}F)$ = 2465 Hz), –46.1 (m, 1F, $^{2}J(^{19}F,^{19}F)$ = 154; 110 Hz, $^{1}J(^{125}Te,^{19}F)$ = 2473 Hz), –48.0 (m, 2F, $^{2}J(^{19}F,^{19}F)$ = 110; 134 Hz, $^{1}J(^{125}Te,^{19}F)$ = 3306 Hz) ppm.

^{29}Si{^{1}H} NMR (80 MHz, CD_2Cl_2, 23 °C): δ = –28.9 (s) ppm.

Synthesis of Ag[*cis*-$PhTeF_4O$] (**3**)

The equimolar amount of AgF (0.41 g, 3.24 mmol) was added to a solution of *cis*-$PhTeF_4OSiMe_3$ (1.20 g, 3.24 mmol) in CH_2Cl_2 (10 mL). The reaction mixture was stirred in the dark at room temperature overnight. After removal of the volatiles under reduced pressure a colourless solid was obtained, which was identified as compound **3** (1.05 g, 2.60 mmol, 80% yield).

^{1}H NMR (400 MHz, CD_3CN, 23 °C): δ = 8.07–7.81 (m, 2H, *o*-H), 7.60–7.47 (m, 3H, *p*-H, *m*-H) ppm.

^{19}F NMR (377 MHz, CD_3CN, 23 °C): δ = –23.3 (dt, 1F, $^{2}J(^{19}F,^{19}F)$ = 148; 123 Hz, $^{1}J(^{125}Te,^{19}F)$ = 3193 Hz), –28.3 (m, 1F, $^{2}J(^{19}F,^{19}F)$ = 123; 146 Hz, $^{1}J(^{125}Te,^{19}F)$ = 2712 Hz), –43.5 (t, 2F, $^{2}J(^{19}F,^{19}F)$ = 123 Hz, $^{1}J(^{125}Te,^{19}F)$ = 2920 Hz) ppm.

IR (ATR, 25 °C): $\tilde{\nu}$ = 3069 (w, C–H), 1476 (m, Ph-ring), 1446 (m, Ph-ring), 993 (m), 921 (m), 746 (m), 677 (s, O–Te–F), 634 (s, Te–F), 458 (s) cm^{-1}.

Synthesis of [PPh_4][*cis*-$PhTeF_4O$] (**4**)

The equimolar amount of [PPh_4]Cl (0.14 g, 0.37 mmol) was added to a suspension of Ag[*cis*-$PhTeF_4O$] (0.15 g, 0.37 mmol) in CH_2Cl_2 (10 mL). The reaction mixture was stirred for 15 min. After filtering the solution to separate the insoluble AgCl, the solvent was removed under reduced pressure to afford a colourless solid, which was identified as compound **4** (0.22 g, 0.35 mmol, 93%). Single crystals of **4** suitable for X-ray diffraction were obtained by slow diffusion of a layer of *n*-pentane (2 mL) into a solution of **4** (10 mg) in CH_2Cl_2 (3 mL) at –40 °C.

^{1}H NMR (400 MHz, CD_2Cl_2, 23 °C): δ = 7.99–7.94 (m, 2H, *o*-H), 7.94–7.87 (m, 4H, *p*-H $[PPh_4]^+$), 7.78–7.71 (m, 8H *m*-H $[PPh_4]^+$), 7.65–7.58 (m, 8H, *o*-H $[PPh_4]^+$), 7.46–7.40 (m, 3H, *p*-H, *m*-H) ppm.

^{19}F NMR (377 MHz, CD_2Cl_2, 23 °C): δ = –22.3 (dt, 1F, $^{2}J(^{19}F,^{19}F)$ = 120; 139 Hz), –27.9 (m, 1F, $^{2}J(^{19}F,^{19}F)$ = 120 Hz), –42.2 (m, 2F, $^{2}J(^{19}F,^{19}F)$ = 114 Hz) ppm.

^{31}P{^{1}H} NMR (104 MHz, CD_2Cl_2, 23 °C): δ = 23.3 (s) ppm.

^{125}Te NMR (126 MHz, CD_2Cl_2, 22 °C): δ = 737 (m) ppm.

IR (ATR, 25 °C): $\tilde{\nu}$ = 3055 (w, C−H), 1482 (w, Ph-ring), 1437 (m, Ph-ring), 1107 (s), 996 (m), 826 (m), 751 (m), 721(s), 689 (s, O−Te−F), 588 (s, Te−F), 577 (s, Te−F), 523 (s), 467 (s) cm^{-1}.

Synthesis of *trans*-$(C_6F_5)_2TeF_4$ (**5**)

$(C_6F_5)_2Te$ (1.66 g, 3.60 mmol), trichloroisocyanuric acid (5.00 g, 21.5 mmol) and potassium fluoride (5.00 g, 86.1 mmol) were suspended in MeCN (60 mL) in a Schlenk flask. After addition of trifluoroacetic acid (28 µL, 0.36 mmol), the reaction mixture was stirred overnight at room temperature. The colourless suspension was filtered and the solid residue washed with MeCN (2×50 mL). The solvent of the filtrate was evaporated to dryness. Extraction of the obtained pale yellow solid with *n*-hexane (3×50 mL) and subsequent removal of the solvent under reduced pressure rendered a colourless solid, which was identified as **5** (1.64 g, 3.05 mmol, 85% yield). Single crystals of **5** suitable for X-ray diffraction were obtained by cooling a saturated solution of **5** in *n*-hexane to –40 °C.

^{13}C{^{19}F} NMR (100 MHz, CD_3CN, 22 °C): δ = 146.5 (s, *o*-C), 145.9 (s, *p*-C), 138.7 (s, *m*-C), 117.3 (s, *ipso*-C) ppm.

^{19}F NMR (377 MHz, CD_3CN, 22 °C): δ = −21.4 (quint, 4F, $^{4}J(^{19}F,^{19}F_o)$ = 19 Hz, $^{1}J(^{125}Te,^{19}F)$ = 3104 Hz, Te−F), −130.2 (m, 4F, $^{3}J(^{19}F_o,^{19}F_m)$ = 20 Hz, $^{3}J(^{125}Te,^{19}F_o)$ = 88 Hz, *o*-F), −143.8 (m, 2F, $^{4}J(^{19}F_o,^{19}F_p)$ = 8.3 Hz, *p*-F), −158.8 (m, 4F, $^{3}J(^{19}F_p,^{19}F_m)$ = 19 Hz, *m*-F) ppm.

^{125}Te NMR (126 MHz, CD_3CN, 22 °C): δ = 770 (m, $^{1}J(^{125}Te,^{19}F)$ = 3090 Hz, $^{3}J(^{125}Te,^{19}F_o)$ = 80 Hz, $^{4}J(^{125}Te,^{19}F_m)$ = 47 Hz, $^{5}J(^{125}Te,^{19}F_p)$ = 10 Hz) ppm.

IR (ATR, 25°C): $\tilde{\nu}$ = 1739 (w), 1639 (m, C–C), 1495 (s, C_6F_5-ring), 1292 (m), 1091 (s, C–F), 983 (s, C–F), 812 (m, C_6F_5-ring), 722 (w), 651 (s, Te–F), 493 (w) cm^{-1}.

MS (ESI+): *m/z*: 540.7 $[(C_6F_5)_2TeF_4]^+$.

Elemental analysis calcd. (%) for $C_{12}F_{14}Te$: C 26.8; found: C 26.8.

Synthesis of K[*trans*-$(C_6F_5)_2TeF_3O$] (**6**)

trans-$(C_6F_5)_2TeF_4$ (0.50 g, 0.93 mmol) was dissolved in a MeCN/H_2O mixture (9:1 V/V, 50 mL) containing potassium fluoride (0.28 g, 4.82 mmol). After stirring overnight at 50 °C, the reaction mixture was dried with $MgSO_4$, filtered, and the solvent was evaporated under reduced pressure. The resulting residue was washed with CH_2Cl_2 (20 mL) and the solvent removed under reduced pressure, rendering a colourless solid, which was identified as compound **6** (0.50 g, 0.87 mmol, 94% yield). Single crystals of **6** suitable for X-ray diffraction were obtained by slow gas diffusion of Et_2O (4 mL) into a solution of **6** (10 mg) in MeCN (3 mL) at –40 °C.

$^{13}C\{^{19}F\}$ NMR (100 MHz, CD_3CN, 22 °C): δ = 142.3 (s, *o*-C), 139.2 (s, *p*-C), 134.0 (s, *m*-C), 113.6 (s, *ipso*-C) ppm.

^{19}F NMR (377 MHz, MeCN, ext. acetone-d_6, 22 °C): δ = 32.3 (tquint, 1F, $^4J(^{19}F,^{19}F_o)$ = 20 Hz, $^2J(^{19}F,^{19}F)$ = 104 Hz, $^1J(^{125}Te,^{19}F)$ = 2412 Hz), −18.8 (dquint, 2F, $^4J(^{19}F,^{19}F_o)$ = 18 Hz, $^1J(^{125}Te,^{19}F)$ = 2471 Hz), −129.9 (m, 4F, $^3J(^{19}F_o,^{19}F_m)$ = 20 Hz, *o*-F), −152.1 (m, 2F, $^4J(^{19}F_o,^{19}F_p)$ = 5 Hz, *p*-F), −161.7 (m, 4F, $^3J(^{19}F_p,^{19}F_m)$ = 19 Hz, *m*-F) ppm.

^{125}Te NMR (126 MHz, CD_3CN, 22 °C): δ = 726 (m, $^1J(^{125}Te,^{19}F)$ = 2512 Hz) ppm.

IR (ATR, 25°C): $\tilde{\nu}$ = 1725 (w), 1637 (m, C–C), 1483 (s, C_6F_5-ring), 1285 (m), 1090 (s, C–F), 977 (s, C–F), 828 (m, C_6F_5-ring), 721 (w), 620 (m, O–Te–F), 596 (s, Te–F) cm^{-1}.

MS (ESI−): *m/z*: 536.9 $[(C_6F_5)_2TeF_3O]^-$.

Elemental analysis calcd. (%) for $C_{12}F_{13}KOTe$: C 25.1; found: C 25.4.

Synthesis of *trans*-$(C_6F_5)_2TeF_3OH$ (**7**)

$K[(C_6F_5)_2TeF_3O]$ (150 mg, 0.26 mmol) was placed in a PFA tube equipped with a stir bar and connected to a stainless steel valve. After cooling to −196°C, *a*HF (1 mL) was condensed into the tube and the resulting suspension was stirred for 15 min at room temperature. All volatiles were evaporated through soda lime scrubbers to remove the unreacted *a*HF and the obtained residue was extracted with CH_2Cl_2 (10 mL). Removal of the solvent under reduced pressure afforded a colourless solid, which was identified as **7** (107 mg, 0.20 mmol, 77% yield).

^{1}H NMR (400 MHz, CD_2Cl_2, 22 °C): δ = 5.76 (br s, OH) ppm.

^{13}C{^{19}F} NMR (100 MHz, CD_2Cl_2, 22 °C): δ = 146.9 (s, *o*-C), 140.0 (s, *p*-C), 138.6 (s, *m*-C), 120.3 (s, *ipso*-C) ppm.

^{19}F NMR (377 MHz, CD_2Cl_2, 22 °C): δ = 2.1 (tquint, 1F, $^4J(^{19}F,^{19}F_o)$ = 20 Hz, $^2J(^{19}F,^{19}F)$ = 54 Hz, $^1J(^{125}Te,^{19}F)$ = 3013 Hz), −26.1 (dquint, 2F, $^4J(^{19}F,^{19}F_o)$ = 19 Hz, $^1J(^{125}Te,^{19}F)$ = 2817 Hz), −129.0 (m, 4F, $^3J(^{19}F_o,^{19}F_m)$ = 20 Hz, *o*-F), −144.1 (m, 2F, $^4J(^{19}F_o,^{19}F_p)$ = 7 Hz, *p*-F), −158.0 (m, 4F, $^3J(^{19}F_p,^{19}F_m)$ = 19 Hz, *m*-F) ppm.

^{125}Te NMR (126 MHz, CD_2Cl_2, 22 °C): δ = 756 (dtm, $^1J(^{125}Te,^{19}F)$ = 3030 Hz, $^1J(^{125}Te,^{19}F)$ = 2838 Hz) ppm.

IR (ATR, 25°C, Figure S15): $\tilde{\nu}$ = 3493 (w, O–H), 1639 (m), 1518 (s), 1485 (s, C_6F_5-Ring), 1397 (m), 1093 (s, C–F), 976 (s, C–F), 810 (m, C_6F_5-Ring), 723 (w), 685 (m), 649 (s, Te–F), 624 (m, O–Te–F), 550 (s, Te–F) cm^{-1}.

MS (ESI−): *m/z*: 1070.7 $[((C_6F_5)_2TeF_3O)_2H]^-$, 536.9 $[(C_6F_5)_2TeF_3O]^-$.

Elemental analysis calcd. (%) for $C_{12}F_{13}HOTe$: C 26.3 H 0.37; found: C 26.5 H 0.47.

2 NMR Spectra

cis-$PhTeF_4OH$ (**1**)

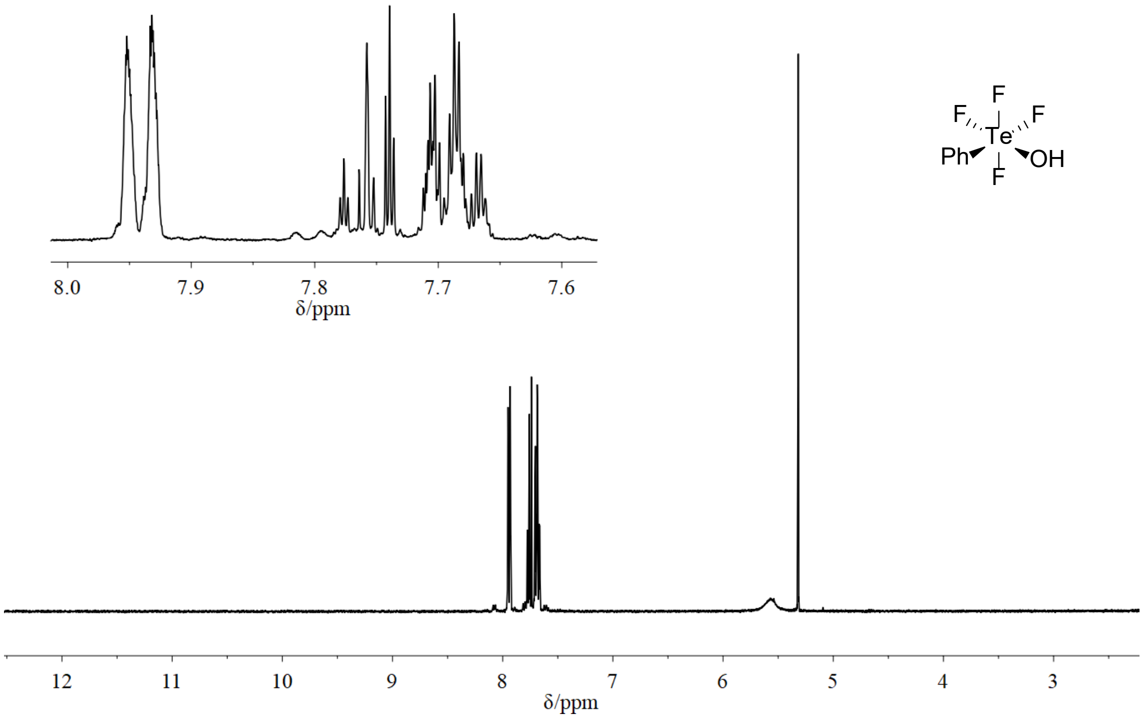

Figure S1. ^{1}H NMR spectrum (400 MHz, CD_2Cl_2, 23 °C) of *cis*-$PhTeF_4OH$ (**1**).

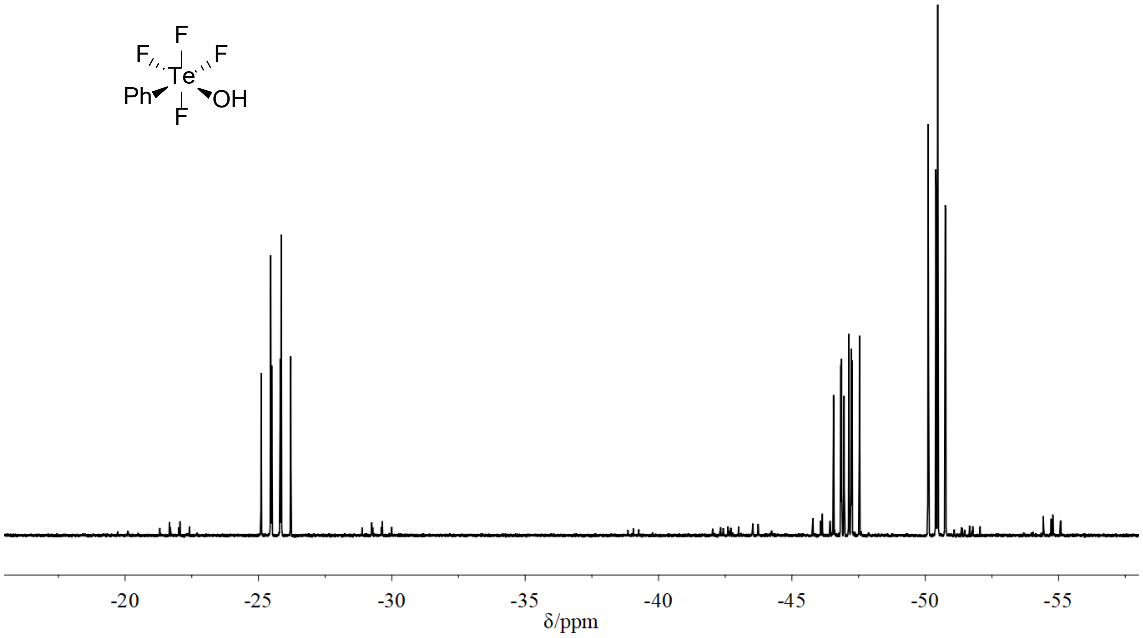

Figure S2. ^{19}F NMR spectrum (377 MHz, CD_2Cl_2, 23 °C) of *cis*-$PhTeF_4OH$ (**1**).

cis-$PhTeF_4OSiMe_3$ (**2**)

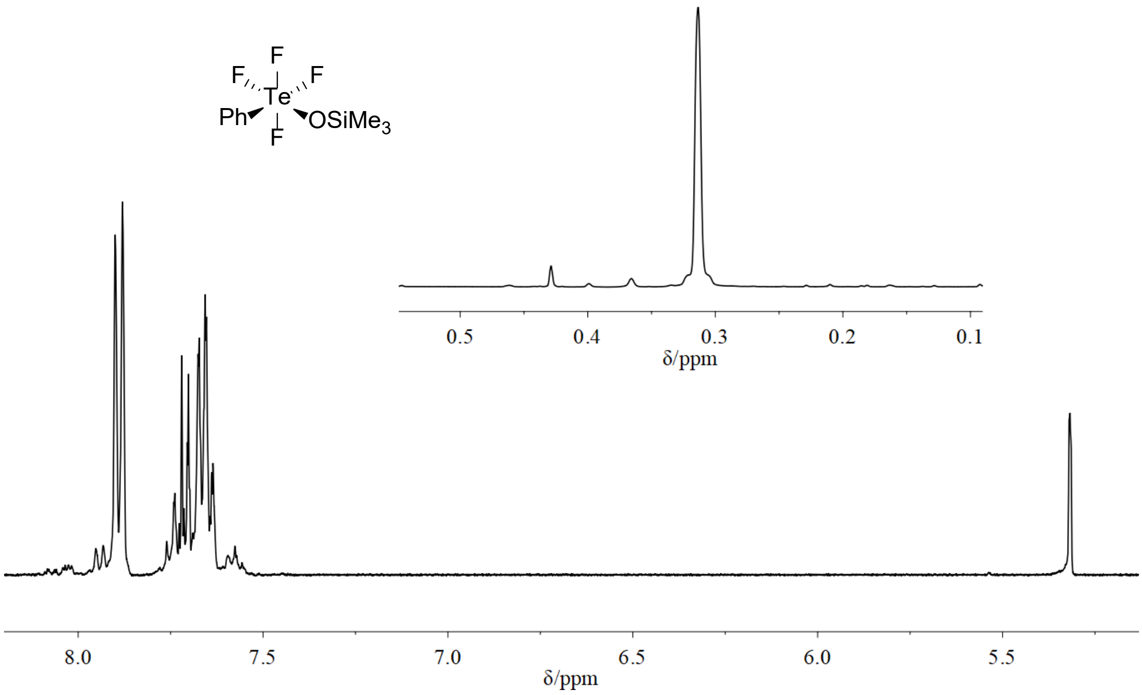

Figure S3. ^{1}H NMR spectrum (400 MHz, CD_2Cl_2, 23 °C) of *cis*-$PhTeF_4OSiMe_3$ (**2**).

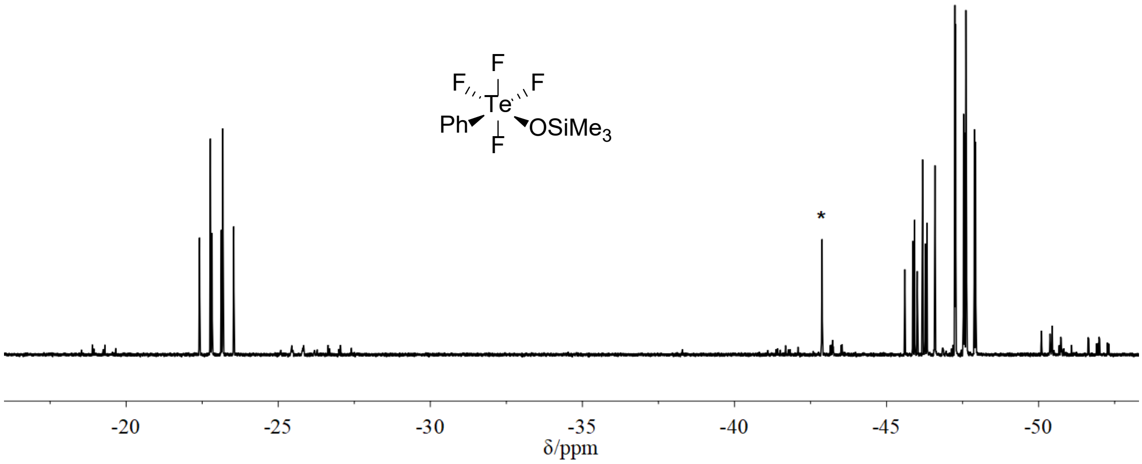

Figure S4. ^{19}F NMR spectrum (377 MHz, CD_2Cl_2, 23 °C) of *cis*-$PhTeF_4OSiMe_3$ (**2**). The marked signal (*) denotes an unidentified species.

Ag[*cis*-PhTeF$_4$O] (**3**)

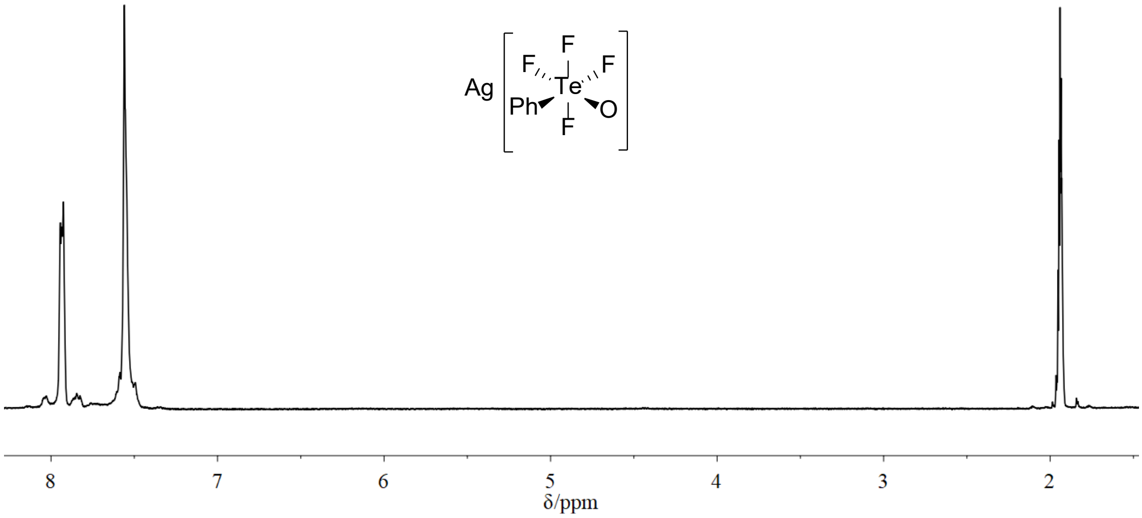

Figure S5. ^{1}H NMR spectrum (400 MHz, CD$_3$CN, 23 °C) of Ag[*cis*-PhTeF$_4$O] (**3**).

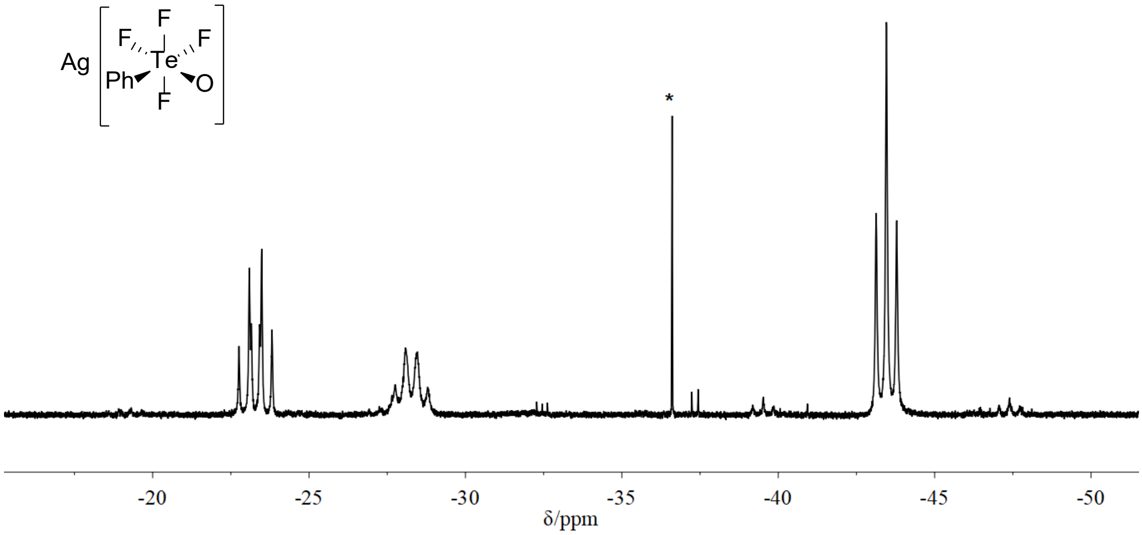

Figure S6. ^{19}F NMR spectrum (377 MHz, CD$_3$CN, 23 °C) of Ag[*cis*-PhTeF$_4$O] (**3**) The marked signal (*) denotes an unidentified species.

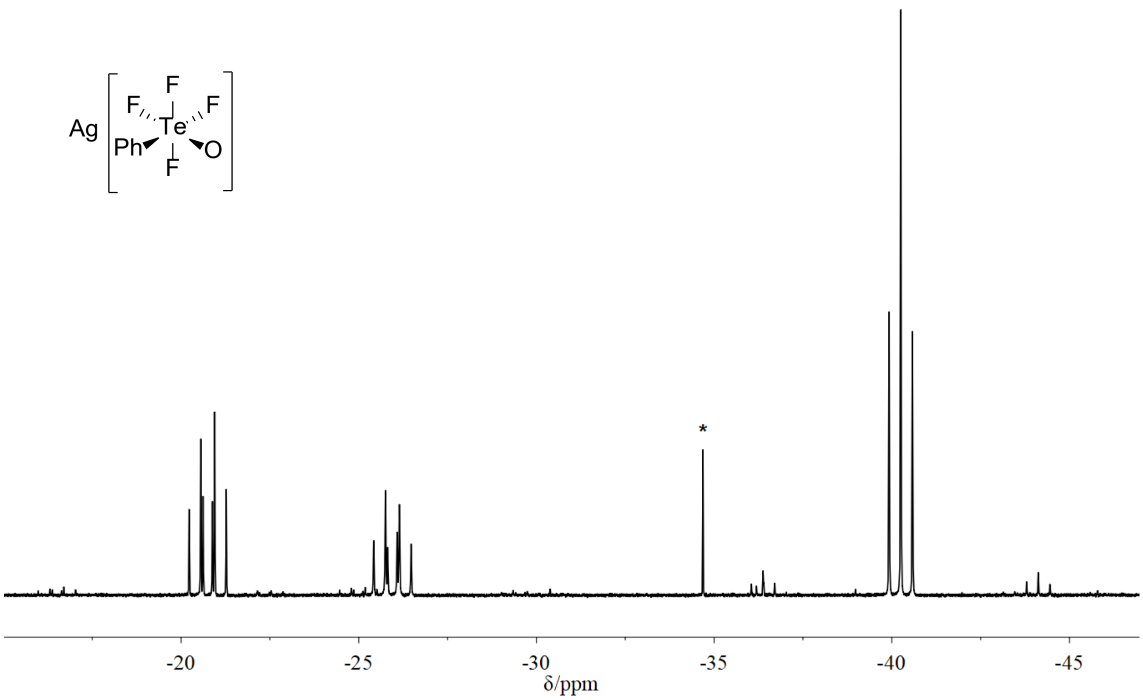

Figure S7. ^{19}F NMR spectrum (377 MHz, CD_3CN, 23 °C) of Ag[*cis*-$PhTeF_4O$] (**3**) after addition of 0.1 mL of pyridine.

[PPh_4][*cis*-$PhTeF_4O$] (**4**)

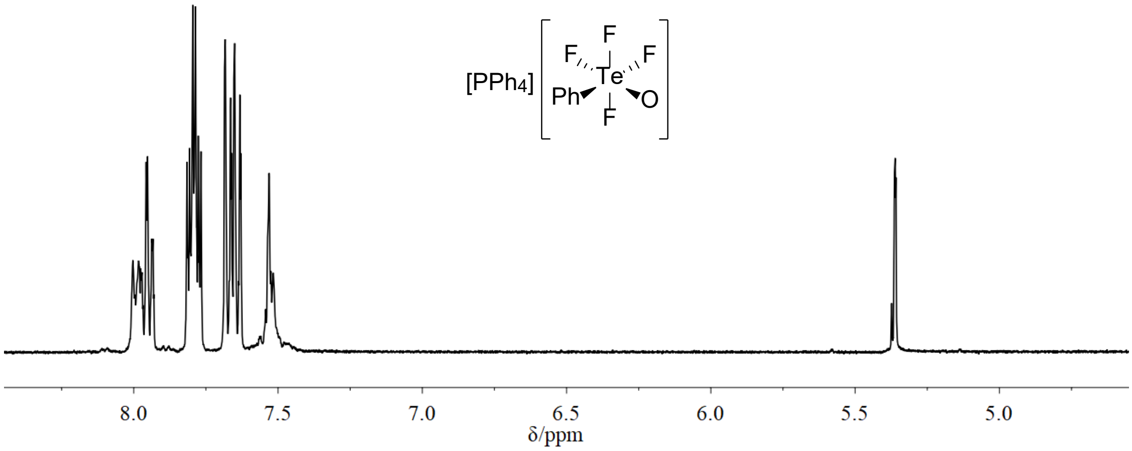

Figure S8. ^{1}H NMR spectrum (400 MHz, CD_2Cl_2, 23 °C) of [PPh_4][*cis*-$PhTeF_4O$] (**4**).

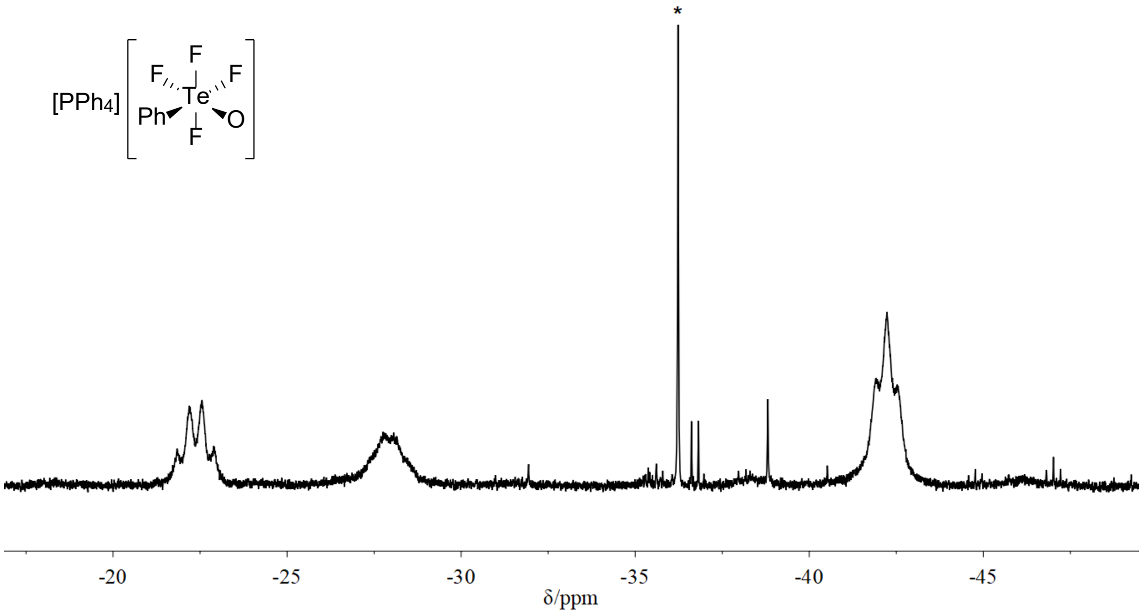

Figure S9. ^{19}F NMR spectrum (377 MHz, CD_2Cl_2, 23 °C) of [PPh_4][*cis*-$PhTeF_4O$] (**4**) The marked signal (*) denotes an unidentified species.

trans-$(C_6F_5)_2TeF_4$ (**5**)

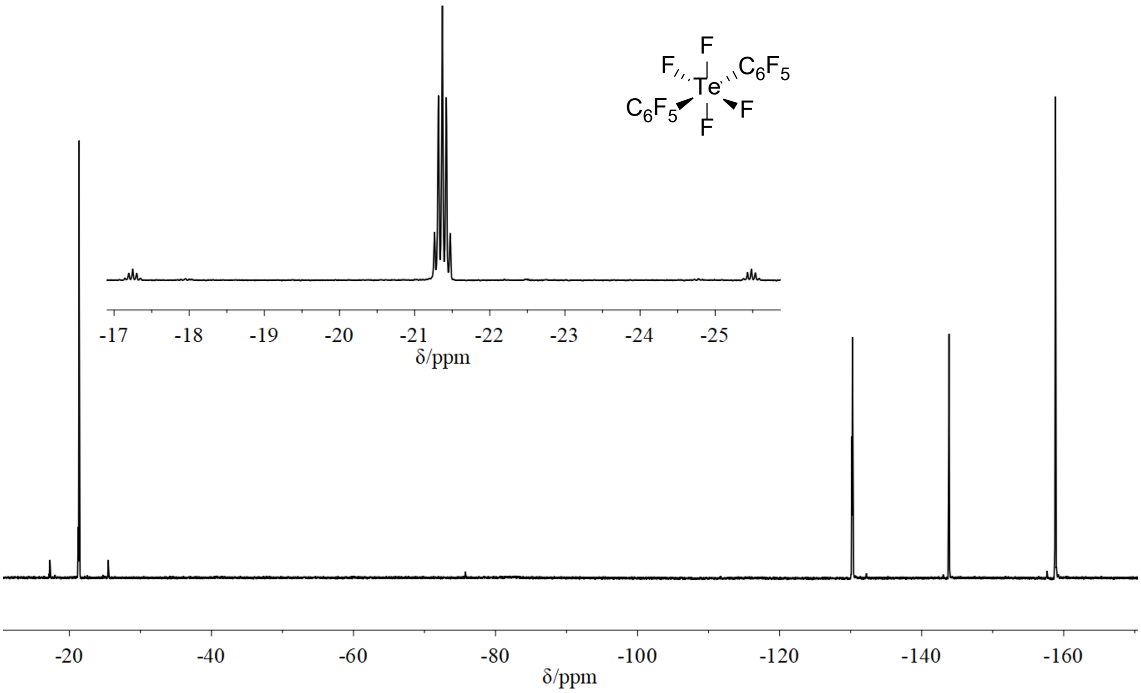

Figure S10. ^{19}F NMR spectrum (377 MHz, CD_3CN, 22 °C) of *trans*-$(C_6F_5)_2TeF_4$ (**5**).

K[*trans*-$(C_6F_5)_2TeF_3O$] (**6**)

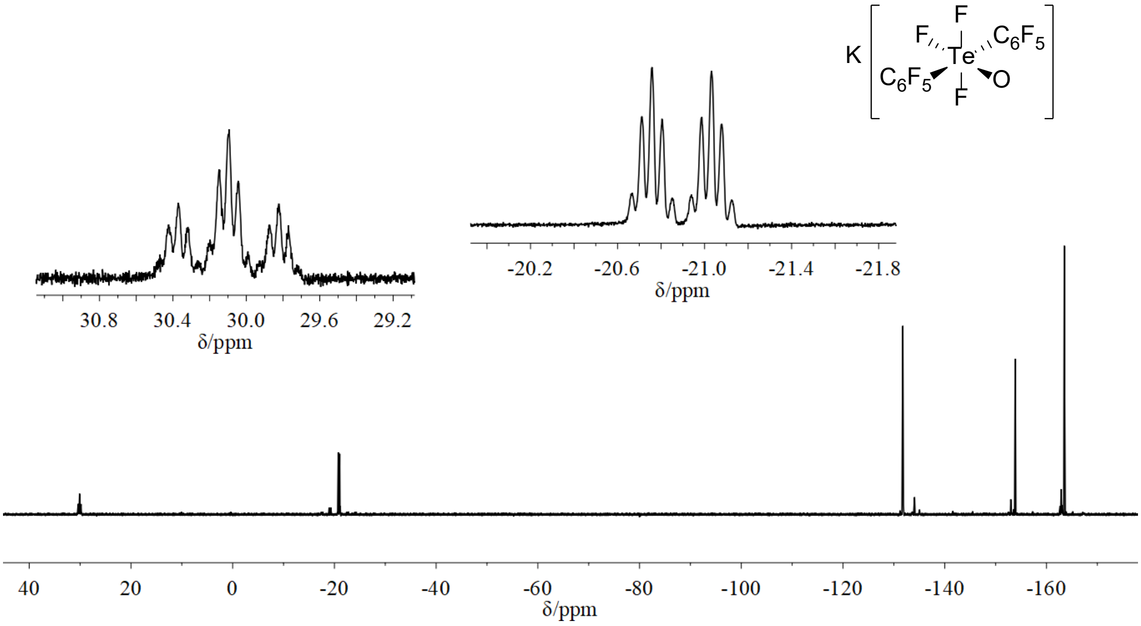

Figure S11. ^{19}F NMR spectrum (377 MHz, MeCN, ext. acetone-d_6, 22 °C) of K[*trans*-$(C_6F_5)_2TeF_3O$] (**6**).

trans-(C_6F_5)$_2$TeF$_3$OH (**7**)

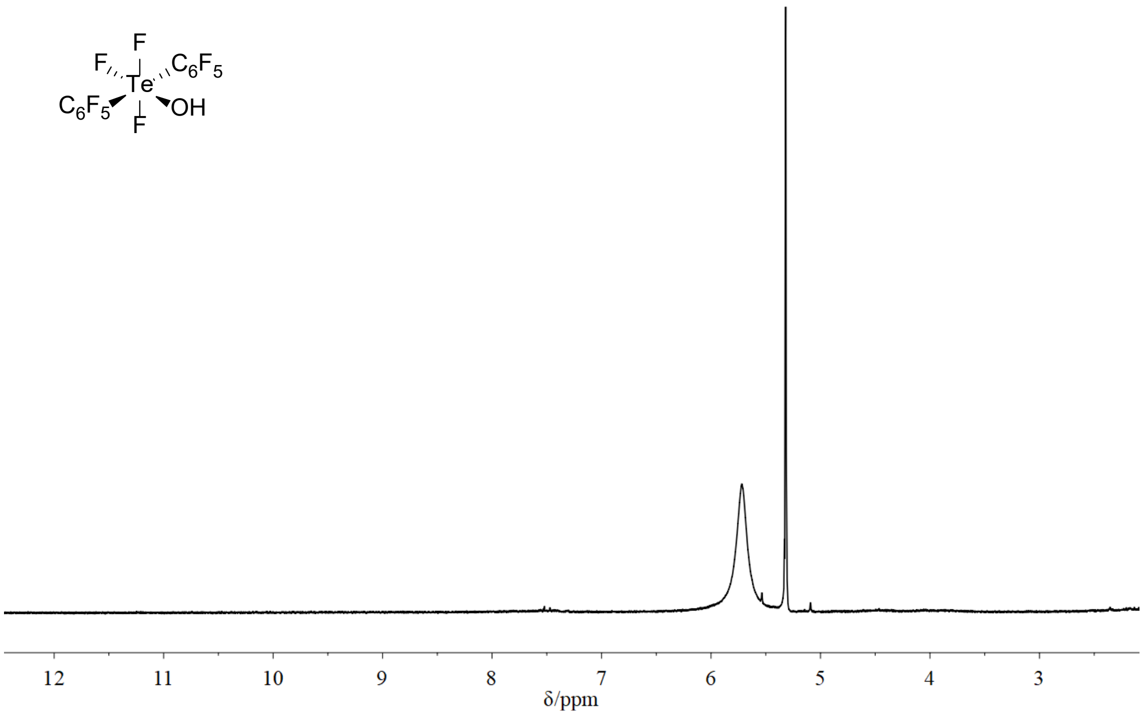

Figure S12. ^{1}H NMR spectrum (400 MHz, CD_2Cl_2, 22 °C) of *trans*-(C_6F_5)$_2$TeF$_3$OH (**7**).

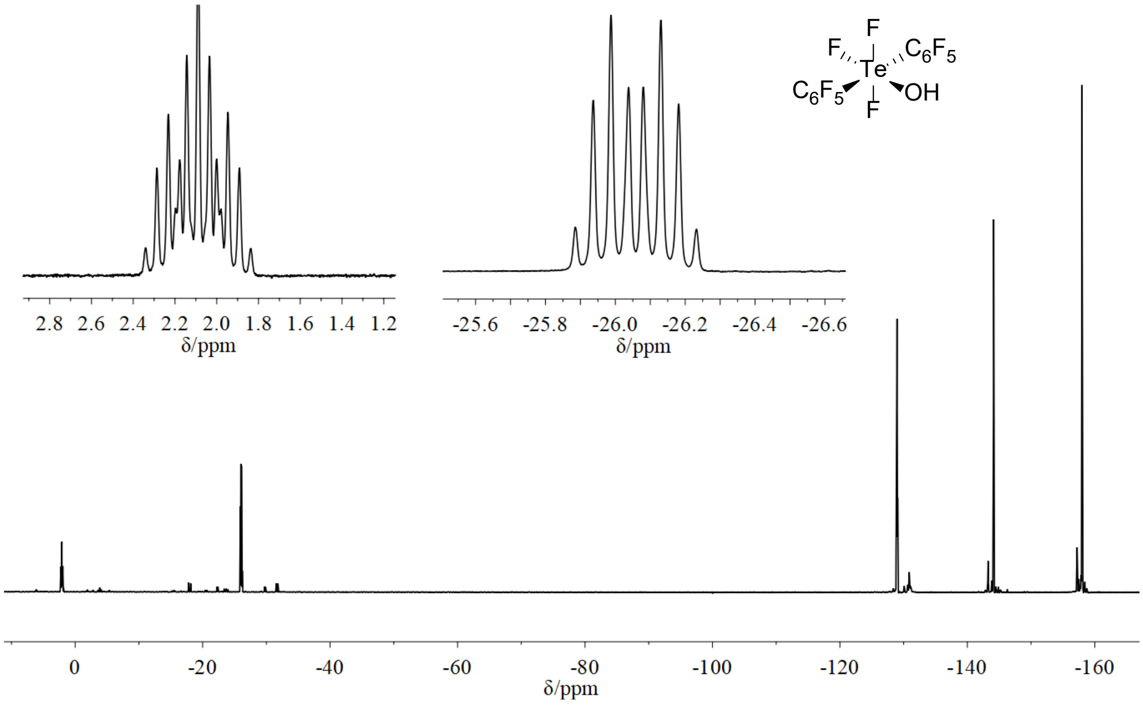

Figure S13. ^{19}F NMR spectrum (377 MHz, CD_2Cl_2, 23 °C) of *trans*-(C_6F_5)$_2$TeF$_3$OH (**7**).

3 IR Spectra

cis-$PhTeF_4OH$ (**1**)

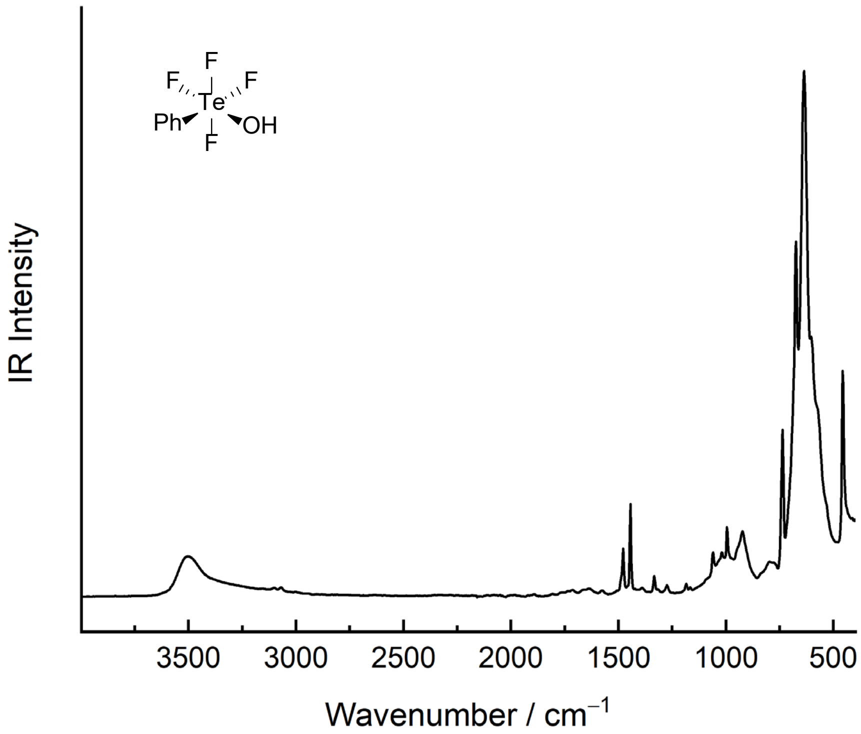

Figure S14. IR spectrum of compound *cis*-$PhTeF_4OH$ (**1**). The characteristic stretching O–H vibration can be observed at 3502 cm^{-1}.

trans-$(C_6F_5)_2TeF_3OH$ (**7**)

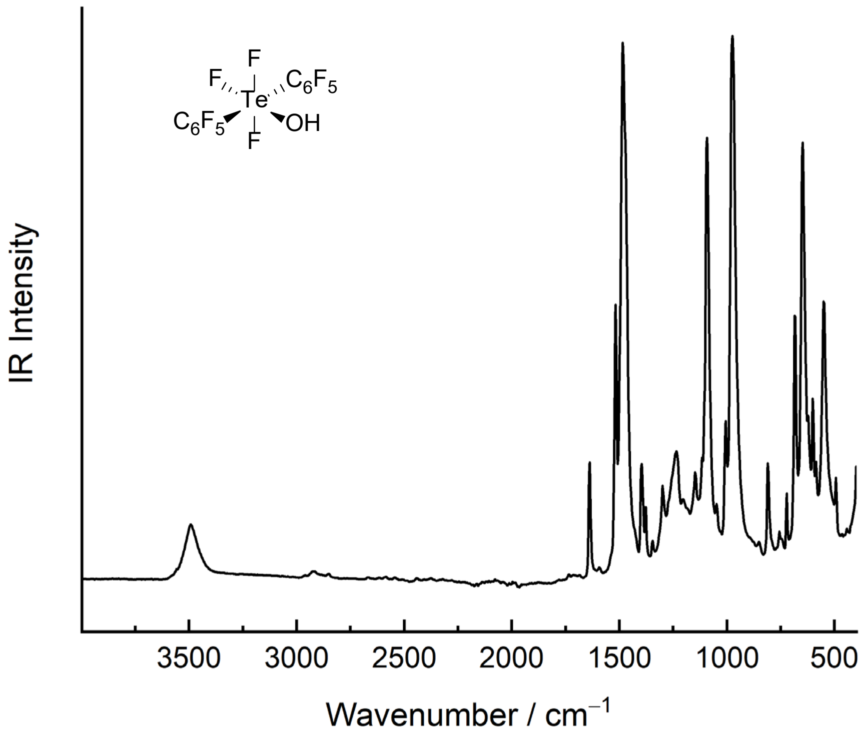

Figure S15. IR spectrum of compound *trans*-$(C_6F_5)_2TeF_3OH$ (**7**). The characteristic stretching O–H vibration can be observed at 3493 cm^{-1}.

4 Crystal Data

Summary of crystal data and structure refinement

Table S1. Crystal data and structure refinement for compound **1**.

Empirical formula	$C_6H_6F_4OTe$
Formula weight	297.71
Temperature/K	102.0
Crystal system	orthorhombic
Space group	*Pbca*
a/pm	858.05(4)
b/pm	1757.59(8)
c/pm	2160.97(9)
α/°	90
β/°	90
γ/°	90
Volume/Å^3	3259.0(3)
Z	16
ρ_{calc} g/cm^3	2.427
μ/mm^{-1}	3.668
F(000)	2208.0
Crystal size/mm^3	0.332 × 0.279 × 0.054
Radiation	MoKα (λ = 0.71073)
2Θ range for data collection/°	3.77 to 56.624
Index ranges	$-11 \leq h \leq 11$, $-23 \leq k \leq 23$, $-28 \leq l \leq 26$
Reflections collected	58950
Independent reflections	4052 [R_{int} = 0.0584, R_{sigma} = 0.0214]
Data/restraints/parameters	4052/0/225
Goodness-of-fit on F^2	1.131
Final R indexes [I>=2σ (I)]	R_1 = 0.0228, wR_2 = 0.0427
Final R indexes [all data]	R_1 = 0.0313, wR_2 = 0.0454
Largest diff. peak/hole / e Å^{-3}	0.54/–0.73
CCDC number	2184677

Table S2. Crystal data and structure refinement for compound **4**.

Empirical formula	$C_{30}H_{25}F_4OPTe$
Formula weight	636.07
Temperature/K	299.0
Crystal system	monoclinic
Space group	$P2_1/c$
a/pm	1120.55(3)
b/pm	1493.02(4)
c/pm	1550.64(5)
α/°	90
β/°	105.2430(10)
γ/°	90
Volume/Å^3	2502.96(13)
Z	4
ρ_{calc}g/cm^3	1.688
μ/mm^{-1}	1.306
F(000)	1264.0
Crystal size/mm^3	0.262 × 0.089 × 0.081
Radiation	MoKα (λ = 0.71073)
2Θ range for data collection/°	3.854 to 56.582
Index ranges	$-14 \le h \le 14$, $-19 \le k \le 19$, $-20 \le l \le 20$
Reflections collected	48355
Independent reflections	6204 [R_{int} = 0.0374, R_{sigma} = 0.0201]
Data/restraints/parameters	6204/0/334
Goodness-of-fit on F^2	1.068
Final R indexes [I>=2σ (I)]	R_1 = 0.0282, wR_2 = 0.0691
Final R indexes [all data]	R_1 = 0.0320, wR_2 = 0.0715
Largest diff. peak/hole / e Å^{-3}	2.98/–1.22
CCDC number	2184735

Table S3. Crystal data and structure refinement for compound **5**.

Empirical formula	$C_{12}F_{14}Te$
Formula weight	537.72
Temperature/K	100.0
Crystal system	orthorhombic
Space group	*Pbca*
a/pm	1133.75(12)
b/pm	889.38(9)
c/pm	1374.71(13)
α/°	90
β/°	90
γ/°	90
Volume/Å^3	1386.2(2)
Z	4
ρ_{calc}g/cm^3	2.577
μ/mm^{-1}	2.314
F(000)	1000.0
Crystal size/mm^3	0.4 × 0.25 × 0.2
Radiation	MoKα (λ = 0.71073)
2Θ range for data collection/°	6.932 to 52.784
Index ranges	$-14 \leq h \leq 14, -11 \leq k \leq 11, -17 \leq l \leq 17$
Reflections collected	65822
Independent reflections	1403 [R_{int} = 0.0266, R_{sigma} = 0.0063]
Data/restraints/parameters	1403/0/124
Goodness-of-fit on F^2	1.129
Final R indexes [I>=2σ (I)]	R_1 = 0.0138, wR_2 = 0.0337
Final R indexes [all data]	R_1 = 0.0141, wR_2 = 0.0339
Largest diff. peak/hole / e Å^{-3}	0.35/–0.44
CCDC number	2184678

Table S4. Crystal data and structure refinement for compound **6**.

Empirical formula	$C_{14}H_3F_{13}KNOTe$
Formula weight	614.87
Temperature/K	100.00
Crystal system	monoclinic
Space group	$P2_1/c$
a/pm	1070.27(5)
b/pm	757.55(3)
c/pm	2181.42(9)
α/°	90
β/°	96.479(2)
γ/°	90
Volume/Å^3	1757.36(13)
Z	4
ρ_{calc}g/cm^3	2.324
μ/mm^{-1}	2.070
F(000)	1160.0
Crystal size/mm^3	0.32 × 0.21 × 0.088
Radiation	MoKα (λ = 0.71073)
2Θ range for data collection/°	5.054 to 55.03
Index ranges	$-13 \leq h \leq 13$, $-9 \leq k \leq 8$, $-28 \leq l \leq 28$
Reflections collected	38987
Independent reflections	4006 [R_{int} = 0.0218, R_{sigma} = 0.0114]
Data/restraints/parameters	4006/0/281
Goodness-of-fit on F^2	1.111
Final R indexes [I>=2σ (I)]	R_1 = 0.0143, wR_2 = 0.0381
Final R indexes [all data]	R_1 = 0.0149, wR_2 = 0.0384
Largest diff. peak/hole / e Å^{-3}	0.37/−0.34
CCDC number	2184734

Table S5. Crystal data and structure refinement for *trans*-$(C_6F_5)_2TeF_2(OH)_2$.

Empirical formula	$C_{14.4}F_{12}H_{6.8}O_{2.8}Te$
Formula weight	580.197
Temperature/K	100.0
Crystal system	monoclinic
Space group	*C*2/*c*
a/pm	1848.85(8)
b/pm	875.91(4)
c/pm	1180.83(5)
α/°	90
β/°	108.111(2)
γ/°	90
Volume/Å^3	1817.53(14)
Z	4
ρ_{calc}g/cm^3	2.120
μ/mm^{-1}	1.794
F(000)	1102.0
Crystal size/mm^3	0.353 × 0.155 × 0.123
Radiation	MoKα (λ = 0.71073)
2Θ range for data collection/°	4.64 to 52.8
Index ranges	$-23 \leq h \leq 23$, $-10 \leq k \leq 10$, $-14 \leq l \leq 14$
Reflections collected	44869
Independent reflections	1864 [R_{int} = 0.0224, R_{sigma} = 0.0072]
Data/restraints/parameters	1864/74/206
Goodness-of-fit on F^2	1.076
Final R indexes [I>=2σ (I)]	R_1 = 0.0212, wR_2 = 0.0564
Final R indexes [all data]	R_1 = 0.0235, wR_2 = 0.0590
Largest diff. peak/hole / e Å^{-3}	1.45/–0.36
CCDC number	2184711

5 Attempted hydrolysis of *trans*-$(C_6F_5)_2TeF_4$ (5)

trans-$(C_6F_5)_2TeF_4$ (20 mg, 38 μmol) was dissolved in a MeCN/H_2O mixture (9:1 V/V, 1 mL) and heated to 50 °C for 4 h. The reaction mixture was extracted with CH_2Cl_2 (3×3 mL). The combined organic phases were dried with $MgSO_4$, filtered, and the solvent was removed under reduced pressure. A colorless solid was obtained (15 mg) and identified as a mixture containing the two isomers of the doubly hydrolysed $(C_6F_5)_2TeF_2(OH)_2$. Single crystals of *trans*-$(C_6F_5)_2TeF_2(OH)_2$ suitable for X-ray diffraction were obtained by cooling a saturated solution of the obtained colorless solid in *n*-hexane to –40 °C.

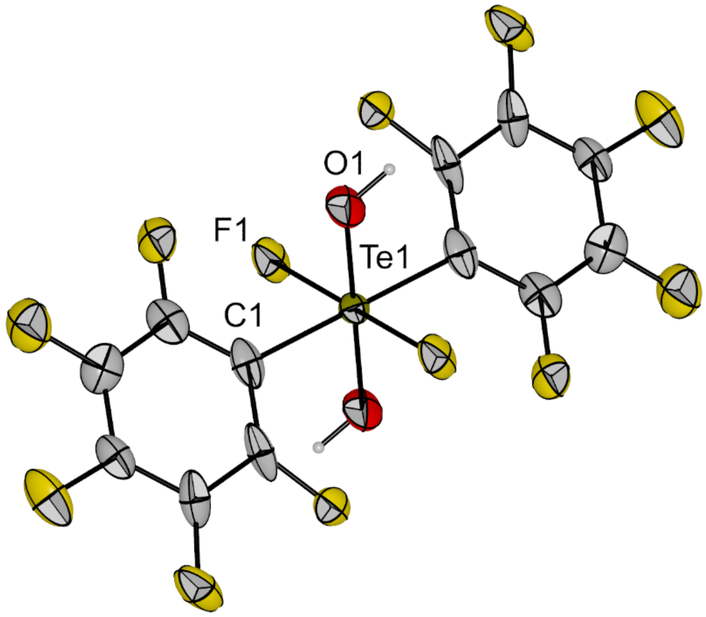

Figure S16. Molecular structure of *trans*-$(C_6F_5)_2TeF_2(OH)_2$ in the solid state. Displacement ellipsoids set at 50% probability. The summary of crystal data and structure refinement appears in Table S5. Selected bond lengths [pm] and angles [°]: Te1–F1 188.9(1), Te1–C1 213.6(2), Te1–O1 188.2(1) C1–Te1–F1 90.3(1), C1–Te1–O1 87.7(1), O1–Te1–F1 88.9(1).

6 Quantum-chemical calculations

The *Turbomole* program[8] was used to perform calculations at the unrestricted Kohn-Sham DFT level, using the BP86 or B3LYP hybrid functional[9–11] (with RI[12]) in conjunction with basis sets def-SV(P) and def2-TZVPP.[13] Minima on potential energy surfaces were characterized by normal mode analysis. Thermochemical data is provided without counterpoise correction but including zero-point energy correction as obtained from harmonic vibrational frequencies.

$HOTeF_5$

Te	0.2953171	0.0226963	0.0078748
F	1.9703345	0.8189488	0.4825549
F	0.3451752	-0.8770939	1.7073183
F	0.3861272	1.0176189	-1.6350632
F	-0.6529239	1.4927568	0.8318399
F	1.1940194	-1.4492153	-0.8172217
O	-1.4158598	-0.8048073	-0.4790520
H	-2.1221896	-0.2209043	-0.0982509

$OTeF_4$

Te	-0.0000001	0.0000000	0.1000779
O	-0.0000000	-0.0000000	1.8666536
F	0.0000001	1.8472003	-0.2168869
F	1.5364612	-0.0000001	-0.9414088
F	-1.5364613	-0.0000001	-0.9414088
F	0.0000001	-1.8472002	-0.2168870

$[OTeF_5]^-$

Te	0.9271398	0.8883497	0.0765649
F	2.7723733	1.3221413	0.5973691
F	0.2840314	1.9220507	1.6199915
F	1.6007790	0.3413339	-1.6875904
F	-0.8897078	0.9312488	-0.6729044
F	1.0142408	2.6674532	-0.7381025
O	0.8351235	-0.7692177	0.8380018

***cis*-$PhTeF_4OH$**

H	0.0137213	0.0073054	5.2511248
C	0.0066153	-0.0004679	4.1488999
C	-0.0875007	-1.2217592	3.4597493
H	-0.1541377	-2.1716571	4.0150486
C	-0.0980964	-1.2480584	2.0531005

H	-0.1709906	-2.1976046	1.5027767
C	-0.0122398	-0.0207069	1.3789628
Te	-0.0308245	-0.0221270	-0.7600110
C	0.0822196	1.2150230	2.0346364
H	0.1465604	2.1561204	1.4689065
C	0.0913544	1.2107018	3.4411166
H	0.1650927	2.1686408	3.9813851
F	-1.9699867	0.1354193	-0.8644157
F	1.9119305	-0.0095122	-0.9029031
O	-0.1051298	-1.9969877	-0.8655821
F	0.0420164	1.9155874	-0.8790781
F	-0.0506750	-0.0363746	-2.6975112
H	-0.1259155	-2.2275458	-1.8262348

$PhTeF_3O$

Te	0.0684967	0.0453848	0.0404214
O	0.2776837	-0.4795917	1.7185058
F	-0.1888877	1.9096218	0.4226957
F	1.6008649	0.6539744	-0.8513037
F	0.5234902	-1.6327157	-0.7748683
H	-2.2058569	2.0932838	-0.5260080
C	-2.4800444	1.2412491	-1.1265867
C	-1.6809034	0.1031210	-1.1677641
C	-2.0008056	-1.0149536	-1.9312519
H	-1.3618654	-1.8829085	-1.9429059
C	-3.1676549	-0.9773167	-2.6868325
H	-3.4333477	-1.8343260	-3.2906921
C	-3.9830884	0.1488240	-2.6666750
H	-4.8887318	0.1674266	-3.2582122
C	-3.6417261	1.2512083	-1.8908956
H	-4.2763632	2.1268281	-1.8753968

[*cis*-$PhTeF_4O]^-$

H	-0.0234326	0.0146026	5.2699821
C	-0.0149662	0.0050532	4.1659296
C	-1.2261545	0.0290909	3.4507311
H	-2.1879651	0.0591986	3.9928114
C	-1.2201179	0.0156547	2.0437424
H	-2.1553696	0.0315346	1.4628272
C	0.0063023	-0.0198513	1.3604520
Te	0.0312076	-0.1155880	-0.8129734
C	1.2223155	-0.0407173	2.0630460
H	2.1691495	-0.0551316	1.5007453
C	1.2071502	-0.0300577	3.4700599
H	2.1607932	-0.0458198	4.0268962
F	-1.9545471	-0.2801281	-0.8242103

F	1.9089498	0.5483623	-0.7926689
O	0.4190083	-1.8939012	-1.0523451
F	-0.3984003	1.8224719	-0.6615490
F	-0.0516196	0.3455321	-2.7240226

***cis*-(C_6F_5)TeF_4OH**

F	1.0281216	-0.5098479	2.8112070
C	0.6443471	-0.2423377	1.5671793
C	-0.6941162	0.1048324	1.3080631
F	-1.5709966	0.1680806	2.3088082
C	-1.1000957	0.3883880	-0.0091531
F	-2.3688209	0.7223855	-0.2175658
C	-0.1680157	0.3132058	-1.0620187
Te	-0.7742838	0.7658376	-3.0525336
C	1.1735688	-0.0243760	-0.7989105
F	2.0830955	-0.0905474	-1.7649834
C	1.5791255	-0.3097875	0.5179009
F	2.8446712	-0.6380915	0.7740853
F	-2.4479472	-0.1880806	-2.8560726
F	0.7780478	1.8885963	-3.3452873
O	0.1300819	-0.8391028	-3.7492743
F	-1.7103786	2.3585389	-2.4969837
F	-1.3052136	1.1684719	-4.8678006
H	-0.0108811	-0.8621457	-4.7288205

(C_6F_5)TeF_3O

Te	0.1087013	0.1328311	0.0561079
O	0.2951841	0.5789647	1.7555227
F	0.4561714	1.8393879	-0.7145981
F	1.6230893	-0.4023405	-0.8919095
F	-0.1765586	-1.7324870	0.2984254
F	-2.5077973	1.8761380	0.1706639
C	-2.6478930	1.0246878	-0.8421508
C	-1.6427193	0.1126005	-1.1338025
C	-1.7968826	-0.7786754	-2.1877901
F	-0.8359916	-1.6378408	-2.5044620
C	-2.9597395	-0.7616915	-2.9461467
F	-3.1125506	-1.6025858	-3.9625679
C	-3.9656139	0.1498693	-2.6431014
F	-5.0757187	0.1654753	-3.3646400
C	-3.8153665	1.0466700	-1.5905968
F	-4.7850545	1.9081065	-1.3067241

[*cis*-(C_6F_5)TeF_4O]$^-$

F	0.9826335	-0.5549216	2.7898731
C	0.6265765	-0.2643614	1.5266570

C	-0.7089264	0.0446956	1.2257387
F	-1.6260644	0.0484337	2.2114451
C	-1.0754037	0.3518863	-0.0998229
F	-2.3615656	0.6427327	-0.3094703
C	-0.1267912	0.3450407	-1.1335878
Te	-0.6487500	0.7565366	-3.2371520
C	1.2031781	0.0321836	-0.8148451
F	2.1637221	0.0085865	-1.7437725
C	1.5892010	-0.2704779	0.5052034
F	2.8693524	-0.5633907	0.8000121
F	-2.3801221	-0.1498742	-2.9662884
F	0.8305713	2.0655964	-3.2763774
O	0.2340974	-0.6536699	-3.9977985
F	-1.6069958	2.2832486	-2.4270342
F	-1.3518631	1.5477549	-4.8875802

***trans*-Ph_2TeF_3OH**

H	-0.1980228	-1.6960223	2.2910730
C	-0.3576609	-1.3326030	1.2625369
C	-1.4072312	-1.8576052	0.4890809
H	-2.0722297	-2.6320221	0.9059486
C	-1.6238978	-1.3989250	-0.8233433
H	-2.4451195	-1.7934132	-1.4391748
C	-0.7620938	-0.4152462	-1.3262628
Te	-1.0714717	0.2981648	-3.3371443
C	0.2935173	0.1279758	-0.5811740
H	0.9483951	0.8997249	-1.0118844
C	0.4879049	-0.3442448	0.7298770
H	1.3102016	0.0705451	1.3361892
F	-2.7578101	-0.7176764	-3.4467686
F	0.7049764	1.2016688	-3.3019410
O	-0.1317067	-1.2917750	-4.1571045
H	-0.8197568	-0.5838659	-6.3102287
H	-1.3367054	0.4171818	-8.5687485
C	-1.2439536	0.4283714	-6.3993524
C	-1.5361463	0.9973311	-7.6525925
C	-1.5129591	1.1870585	-5.2520778
C	-2.0787633	2.2912074	-7.7355403
C	-2.0577002	2.4774385	-5.3014922
C	-2.3369603	3.0270385	-6.5660628
H	-2.2598575	3.0344756	-4.3757878
H	-2.7639976	4.0415645	-6.6301302
H	-2.3045508	2.7299773	-8.7214457
H	0.8236759	-1.0394351	-4.1532048
F	-1.9201363	1.8824203	-2.4877041

[*trans*-Ph_2TeF_3O]$^-$

H	-0.1908441	-1.7505251	2.3144119
C	-0.3471780	-1.3695022	1.2896247
C	-1.5128482	-0.6455536	0.9775329
H	-2.2716093	-0.4584242	1.7583491
C	-1.7195789	-0.1566719	-0.3261311
H	-2.6193269	0.4120585	-0.6023370
C	-0.7474722	-0.4011239	-1.3083573
Te	-0.9245744	0.2938420	-3.3675907
C	0.4203893	-1.1212857	-1.0122143
H	1.1487592	-1.2807833	-1.8269185
C	0.6180770	-1.6064786	0.2938420
H	1.5342953	-2.1743246	0.5354693
F	-2.0732824	-1.3005458	-3.7749567
F	-0.2462875	2.0900322	-2.7829981
O	0.6883602	-0.4019832	-3.9614717
H	0.3030379	0.4236868	-6.1292179
H	-0.2278749	1.3807055	-8.4379335
C	-0.6239752	0.9886561	-6.3321261
C	-0.9307981	1.5222855	-7.5975945
C	-1.5244059	1.1737414	-5.2712770
C	-2.1285032	2.2341028	-7.7931376
C	-2.7230426	1.8813697	-5.4505375
C	-3.0210072	2.4120675	-6.7198116
H	-3.3995061	2.0023472	-4.5920168
H	-3.9617773	2.9709274	-6.8716600
H	-2.3682595	2.6526145	-8.7867248
F	-2.7089669	1.0615350	-2.7128472

***trans*-$(C_6F_5)_2TeF_3OH$**

F	-0.0243692	-1.5290082	2.6153443
C	-0.2567036	-1.1789793	1.3528232
C	-1.5183614	-0.6797837	0.9842850
F	-2.4798936	-0.5626441	1.9000436
C	-1.7639272	-0.3102270	-0.3518795
F	-2.9747288	0.1440763	-0.6587986
C	-0.7432306	-0.4227411	-1.3128386
Te	-1.0643620	0.2380090	-3.3361547
C	0.5102906	-0.9346158	-0.9386190
F	1.5035754	-1.0766896	-1.8253309
C	0.7631637	-1.3076693	0.3929167
F	1.9574075	-1.7833025	0.7483676
F	-2.7401565	-0.7460498	-3.3877310
F	0.6659532	1.1644689	-3.2939299
O	-0.1905186	-1.3480231	-4.1557745
F	-1.8884883	1.8262734	-2.5461675

F	0.7005130	0.7131961	-6.0397504
F	0.1670182	1.9759738	-8.3709402
C	-0.5201848	1.2221580	-6.2225006
C	-0.7838655	1.8724266	-7.4413169
C	-1.5182639	1.1280415	-5.2371933
C	-2.0599050	2.4131994	-7.6796500
C	-2.7954155	1.6637170	-5.4836123
C	-3.0666014	2.3110754	-6.7035017
F	-3.7757257	1.5862628	-4.5889119
F	-4.2748541	2.8245365	-6.9359877
F	-2.3129542	3.0263239	-8.8332692
H	0.7846882	-1.1952048	-4.1097621

$(C_6F_5)_2TeF_2O$

Te	-0.3520469	-0.8508231	0.5400608
O	-1.5277570	-1.6796391	1.5758974
F	-0.4445896	0.8818566	1.3867592
F	0.0754668	-2.3451259	-0.6051789
F	-3.0153747	0.6857090	-0.2446308
C	-2.1965786	0.7404557	-1.2901435
C	-0.9405490	0.1500870	-1.2366928
C	-0.1078646	0.2178416	-2.3453516
F	1.1148391	-0.3112143	-2.3161818
C	-0.5213590	0.8637611	-3.5011876
F	0.2821177	0.9381874	-4.5583077
C	-1.7836041	1.4446258	-3.5462218
F	-2.1849276	2.0615147	-4.6488999
C	-2.6256749	1.3850780	-2.4422219
F	-3.8324295	1.9393977	-2.4969406
F	1.5284230	-2.9547707	1.9903551
C	2.2738018	-1.9011580	1.6731006
C	1.7158353	-0.8112339	1.0171345
C	3.6169992	-1.9031148	2.0234971
C	2.5170330	0.2782133	0.7028767
F	4.1476855	-2.9414528	2.6612970
F	2.0213494	1.3256233	0.0451553
C	4.4078957	-0.8053177	1.7061430
C	3.8618327	0.2877262	1.0430553
F	5.6921385	-0.8009871	2.0350182
F	4.6304678	1.3274202	0.7318388

[*trans*-$(C_6F_5)_2TeF_3O]^-$

F	-0.0127632	-1.3820880	2.7046025
C	-0.2426473	-1.1058151	1.4097451
C	-1.5072802	-0.6563567	1.0001437
F	-2.4825847	-0.5030966	1.9147149

C	-1.7418594	-0.3641691	-0.3579989
F	-2.9698608	0.0523247	-0.6740640
C	-0.7275171	-0.5181967	-1.3151556
Te	-1.0113558	-0.0233209	-3.4475590
C	0.5276986	-0.9774138	-0.8897941
F	1.5404162	-1.1499920	-1.7444431
C	0.7801600	-1.2721563	0.4637728
F	1.9897724	-1.7050592	0.8631566
F	-2.8518598	-0.7533774	-3.3749256
F	0.6052760	1.1228447	-3.2943822
O	-0.2354599	-1.5058467	-4.2019802
F	-1.8617248	1.5922146	-2.6364264
F	0.6887201	0.6930537	-6.1542884
F	0.1583530	2.0690427	-8.4182694
C	-0.5321442	1.2201490	-6.2876953
C	-0.7916478	1.9299922	-7.4758957
C	-1.5107545	1.0782498	-5.2931950
C	-2.0560307	2.5047684	-7.6762427
C	-2.7687483	1.6611165	-5.5080050
C	-3.0483748	2.3683810	-6.6936439
F	-3.7616225	1.5718923	-4.6203868
F	-4.2586097	2.9219919	-6.8931106
F	-2.3174007	3.1822569	-8.8070436

Me_3SiF

Si	-0.0430541	0.0000935	-0.0022416
C	-0.0396398	-1.6781919	-0.7318168
C	-0.0379295	1.4709838	-1.0909968
C	-0.0530159	0.2074585	1.8159551
H	0.8456071	-1.7981384	-1.3664674
H	-0.9040342	-1.7913157	-1.3954153
H	-0.054228	-2.4690992	0.0161714
H	-0.0180927	1.2184628	-2.1497985
H	0.8254775	2.1017039	-0.852471
H	-0.9240382	2.0811934	-0.8825614
H	0.8170949	-0.3000938	2.2470126
H	-0.9327501	-0.2928952	2.2359021
H	-0.0508851	1.2508097	2.1268189

$[Me_3Si]^+$

Si	2.1627348	1.5902503	-0.000005
F	3.7834859	1.5901311	0.0000253
C	1.6118297	0.8009966	-1.6014181
C	1.6118028	3.3716991	0.1171485
C	1.6117956	0.5981561	1.4842719
H	1.9804593	1.3594345	-2.4640235

H	0.5218225	0.7683298	-1.6675619
H	1.9806113	-0.223221	-1.6840603
H	1.980439	3.9553023	-0.7286194
H	1.980509	3.8395207	1.0320262
H	0.5218018	3.4453311	0.1220027
H	1.9805096	-0.4280842	1.432049
H	0.5217891	0.5571943	1.5455958
H	1.9804996	1.0388194	2.4125588

7 References

1 T. M. Klapötke, B. Krumm, P. Mayer, K. Polborn and O. P. Ruscitti, *Inorg. Chem.*, 2001, **40**, 5169.
2 D. Bornemann, C. R. Pitts, C. J. Ziegler, E. Pietrasiak, N. Trapp, S. Kueng, N. Santschi and A. Togni, *Angew. Chem. Int. Ed.*, 2019, **58**, 12604.
3 R. K. Harris, E. D. Becker, S. M. Cabral de Menezes, P. Granger, R. E. Hoffman and K. W. Zilm, *Pure Appl. Chem.*, 2008, **80**, 59.
4 G. M. Sheldrick, *Acta Cryst. A*, 2015, **71**, 9.
5 G. M. Sheldrick, *Acta Cryst. C*, 2015, **71**, 3.
6 O. V. Dolomanov, L. J. Bourhis, R. J. Gildea, J. A. K. Howard and H. Puschmann, *J. Appl. Cryst.*, 2009, **42**, 339.
7 K. Brandenburg, *DIAMOND*, Crystal Impact GbR, Bonn, 2014.
8 TURBOMOLE GmbH, *TURBOMOLE V7.3. a development of University of Karlsruhe and Forschungszentrum Karlsruhe GmbH*, 2018.
9 A. D. Becke, *Phys. Rev. A*, 1988, **38**, 3098.
10 C. Lee, W. Yang and R. G. Parr, *Phys. Rev. B*, 1988, **37**, 785.
11 S. H. Vosko, L. Wilk and M. Nusair, *Can. J. Phys.*, 1980, **58**, 1200.
12 M. Sierka, A. Hogekamp and R. Ahlrichs, *J. Chem. Phys.*, 2003, **118**, 9136.
13 F. Weigend and R. Ahlrichs, *Phys. Chem. Chem. Phys.*, 2005, **7**, 3297.
